# The earliest cut marks of Europe: a discussion on hominin subsistence patterns in the Orce sites (Baza basin, SE Spain)

**DOI:** 10.1038/s41598-019-51957-5

**Published:** 2019-10-28

**Authors:** M. Patrocinio Espigares, Paul Palmqvist, Antonio Guerra-Merchán, Sergio Ros-Montoya, José Manuel García-Aguilar, Guillermo Rodríguez-Gómez, Francisco J. Serrano, Bienvenido Martínez-Navarro

**Affiliations:** 1 Departamento de Ecología y Geología, Universidad de Málaga, Campus de Teatinos, 29071 Malaga, Spain; 2 IPHES, Institut Català de Paleoecologia Humana i Evolució Social, C/Marcel.lí Domingo s/n, Campus Sescelades, Edifici W3, 43007 Tarragona, Spain; 3 Area de Prehistoria, Universitat Rovira i Virgili (URV), Avda. Catalunya 35, 43002 Tarragona, Spain; 4 ICREA, Pg. Lluís Companys 23, 08010 Barcelona, Spain

**Keywords:** Palaeontology, Palaeoecology

## Abstract

Ancient evidence of human presence in Europe is recorded in several Early Pleistocene archaeopalaeontological sites from Spain, France and Italy. This is the case of Barranco León (BL) and Fuente Nueva-3 (FN-3), two localities placed near the town of Orce (depression of Baza and Guadix, SE Spain) and dated to ~1.4 Ma. At these sites, huge assemblages of Oldowan tools and evidence of defleshing, butchering and marrow processing of large mammal bones have been recovered together with a deciduous tooth of *Homo* sp. in the case of level BL-D. In this study, we: (i) describe in detail the anthropic marks found in the bone assemblages from these sites; (ii) analyse patterns of defleshment, butchery and marrow processing, based on the modifications identified in the cortical surface of the fossils; and (iii) discuss on the subsistence strategies of the first hominins that inhabited the European subcontinent during Early Pleistocene times.

## Introduction

Ancient evidence of human presence in Europe has been documented in a number of Early Pleistocene sites that preserve assemblages of Oldowan tools, including TE9 level of Atapuerca Sima del Elefante in Spain (~1.2 Ma)^1^, Pirro Nord in Italy (1.7-1.3 Ma)^2^ and Lézignan-la-Cèbe (1.3-1.1 Ma), Vallonnet Cave (1.2-1.1 Ma) and Pont-de-Lavaud (~1.1 Ma) in France^3^. In this context, two archaeopalaeontological localities near the town of Orce (SE Spain), Barranco León (BL) and Fuente Nueva-3 (FN-3), are of the highest relevance, as they provide some of the earliest records of human presence in Western Europe. These sites, dated to ~1.4 Ma^4–8^, preserve evidence of butchery and marrow processing as well as huge assemblages of lithic artifacts of Oldowan tradition together with a human deciduous tooth in the case of BL^8^.

Evidence of butchery in Europe is relatively young if we compare it with the African record, where the oldest cut marks conclusively associated to stone tools at Gona (Ethiopia) are dated to 2.6-2.5 Ma^9^. Other Early Pleistocene localities from East and North Africa that preserve evidence of ancient anthropic activity are Bouri (~2.5 Ma)^10^, Ain Boucherit (2.4-1.9 Ma)^11^, Koobi Fora (~1.9 Ma)^12^, FLK Zinj (Olduvai Gorge, ~1.8 Ma)^13^, and Ain Hanech (1.8 Ma)^14^.

In spite of the finding of butchery marks that can be confidently tracked to the origin of the genus *Homo* or even earlier, the record of these marks is not frequent. For this reason, a number of issues related to patterns of resource exploitation and subsistence strategies in ancient *Homo* are ultimately based on classical ethnographical studies of modern hunter-gatherers^15–21^. However, the results of these studies relate with the ethology of anatomically modern humans, which does not necessarily reflect the behaviour of other early human species less encephalized and with less developed technical skills (e.g., Oldowan tools). Given these limitations, evidence of anthropic activity in sites with chronologies in excess of one million years can provide key information for deciphering the behaviour of early *Homo*.

### The sites of Barranco León and Fuente Nueva-3

**Figure 1:**
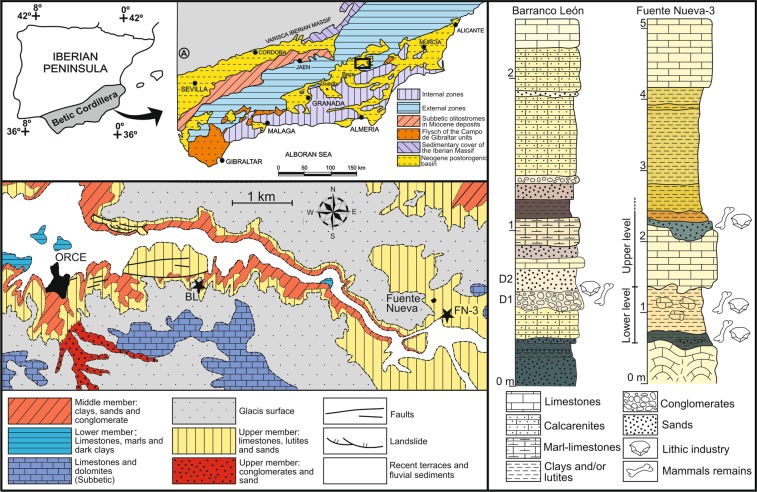
Geographical location and stratigraphic series of Barranco León (BL) and Fuente Nueva-3 (FN-3) sites.

BL and FN-3 lie in the NE sector of the depression of Baza and Guadix (Orce, SE Spain) (Fig. 1), a postorogenic Neogene-Quaternary intramontane basin that covers an area of ~4,000 km^2^ and preserves a thick sedimentary record composed of lacustrine and fluvial deposits^22^. The depression was subject during the Early Pleistocene to intense hydrothermal activity. This was a major determinant in the establishment of biodiversity “hot spots” for the fauna of large mammals, which remains were preserved in abundant palaeontological localities in the vicinities of Orce^22–25^.

The age of the fertile levels of BL and FN-3 has been estimated in 1.43 ± 0.38 Ma and 1.19 ± 0.21 Ma, respectively, using a combined approach based on biostratigraphy, magnetostratigraphy and electron spin resonance (ESR)^6,8^. In addition, an age of 1.50 ± 0.31 Ma has been derived for FN-3 based on cosmogenic nuclides^26^. Moreover, suids are absent from Europe in the biochronological range comprised between the post Tasso Faunal Unit, which marks the base of the Late Villafranchian (~1.8 Ma), and their arrival in Western Europe at layer TE9 from Sima del Elefante (1.22 Ma)^27^. Therefore, their absence from BL and FN-3 suggests that both sites are older than 1.22 Ma.

Research in BL and FN-3 began in the eighties, although anthropic presence at these sites is documented since the nineties^4,28^ by the occurrence of huge tool assemblages, which are associated to an important record of 18 species of large mammals. Remains of small mammals (15 spp., including a porcupine) and herpetofauna (23 taxa) are also well preserved and there are some avian fossils (see details in Supplementary Table S1). Up to the year 2015, the lithic assemblage of BL and FN-3 is composed of ~3,500 Mode 1 stone artifacts (2,124 from BL-D and 1,367 from FN-3 levels), mainly flakes and debris, made on flint and limestones that outcrop in the surroundings of the sites^29,30^. It is worth noting that although evidence of anthropic activity on the bones of large mammals from these sites has been reported in a number of publications^4,7,8,28^, this is the first comprehensive study on them.

The estratigraphy of BL spans the middle terrigenous member and the upper silty calcareous member of the Baza Formation^31^, which are dominated by limestones, sandstones, carbonate silts and dark mudstones deposited in a lacustrine system with an alternation of oligo- to mesosaline waters^28,32^. The excavated layers show sediments associated with a swampy environment, except level D, which shows fluvial features and encloses most of the archaeopalaeontological assemblage^8^. This level is divided in two sub-layers, D1 and D2 (Fig. 1). In the case of FN-3, the semi-horizontal stratigraphy of the site shows three sedimentary cycles deposited in a lutitic-carbonate lacustrine to swampy environment, each with limestones at the top of the sequence separated by clays, fine sands and marly lutites. Subaerial pedogenesis is evident in all stratigraphic levels, including the archaeological ones. The fertile stratum comprises six layers, which group in two main archaeological levels: the Lower Level (layers 1–3) and the Upper Level (layers 4–6)^4,7,28^.

## Results

As explained in the section of Material and Methods, the huge faunal assemblages of BL and FN-3 are composed of many skeletal remains (>6.500 in BL and >9.000 in FN-3). However, many of these remains are badly preserved and need restoration, which precludes their taphonomic analysis. For this reason, the search of modification traces in the cortical surface of bones was focused on those subsamples of skeletal remains that are well preserved and restored, which represent 64.7% of the assemblage from BL and 42.6% in the case of FN-3.

Fossil bones from 18 species of large mammals distributed among eleven families (Felidae, Hyaenidae, Canidae, Ursidae, Mustelidae, Elephantidae, Rhinocerotidae, Equidae, Hippopotamidae, Bovidae, and Cervidae) were identified in BL and FN-3 (Supplementary Table S1). Of these taxa, carnivores represent 3.4% of the number of identifiable specimens (NISP) preserved at level BL-D and 6.5% in the case of the FN-3 levels. In the latter site, carnivores are particularly abundant in the Upper Level, which preserves 81.4% of their remains (Supplementary Table S2). High numbers of skeletal elements (>70% in both sites) cannot be taxonomically classified and are grouped in the category Mammal indet. Turtle remains are very abundant in level BL-D (NISP = 306), while they are less represented in the FN-3 levels (NISP = 60). These remains are composed of small carapace fragments from three testudinoids (Supplementary Table S1), two aquatic turtles and one terrestrial tortoise^33^.

Isolated teeth predominate in BL and FN-3, representing up to 50% of the identifiable remains unearthed from both sites (Supplementary Tables S3 and S4). Among postcranial elements, the bones of the stylopodium and zeugopodium are the most frequently preserved (934 in BL and 931 in FN-3, respectively). In contrast, although autopodial elements are comparatively more abundant in the skeleton, they are less represented in the assemblages by identifiable fragments or complete bones (341 in BL and 432 in FN-3, respectively). The elements of the axial skeleton are comparatively scarce and consist of rib fragments, vertebrae, scapuli, and pelves (Supplementary Tables S3 and S4). Hyena coprolites are particularly abundant in FN-3 (NISP = 157, 1.7% of the assemblage), being preserved almost exclusively in the Upper Level. In contrast, they are less recorded in BL (NISP = 31, 0.5%).

Equids predominate in both sites, followed by hippos and cervids in BL (Supplementary Table S5) and by proboscideans and hippos in FN-3 (Supplementary Table S7). Adult specimens dominate age groups in both assemblages (Supplementary Tables S6 and S8), except in the case of megamammals: proboscideans are better represented by immature individuals, while hippos show minimal numbers of individuals (MNI) that are similar for adults and immatures. The elephant record concentrates in the Upper Level of FN-3 and the count of minimal number of elements (MNE) is much lower than the NISP one, which is due to the abundance of ivory fragments (Supplementary Table S7). Ursids and canids predominate among carnivores in both sites (for their abundance among levels, see Supplementary Tables S5 and S7) and their remains are exclusively from adult individuals (Supplementary Tables S6 and S8). The rate of skeletal survival in both localities evidences an important preservational bias: animals of large and very large size, the size categories better represented, show a high abundance of appendicular bones, mandibles and skulls, and a low representation of elements from the axial skeleton (Supplementary Tables S9 and S10).

**Figure 2:**
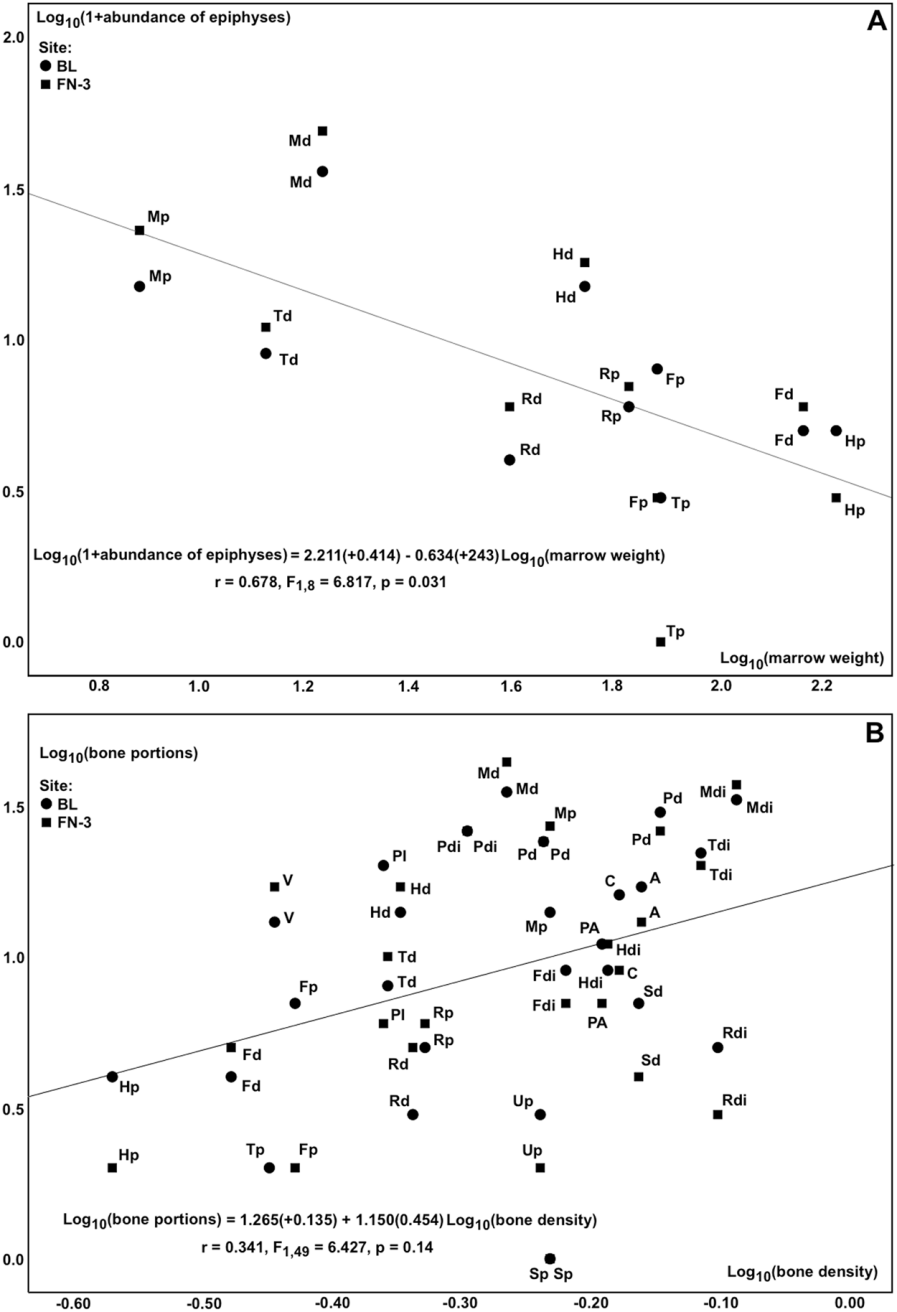
(**A**) Relationship between the abundance of bone epiphyses (p: proximal, d: distal) of major limb bones (H: humerus, R: radius, F: femur, T: tibia, M: metapodial) of ungulates and their estimated marrow weights (mean for horse^34^ and bison^35^). (**B**) Relationship between the abundance of skeletal remains and bone portions (V: vertebrae, S: scapulae, H: humerus, R: radius, U: ulnae, PA: pelvis acetabulum, PI: pelvis ischium/ilium, F: femur, T: tibia, A: astragalus, C: calcaneum, M: metapodial, P: phalanx, p: proximal, di: diaphysis, d: distal) and their estimated mineral densities (mean for horse, gnu and raindeer^36^). In both analyses, data on bone survival for skeletal elements unearthed from BL and FN-3 were considered separately.

An analysis of survival of bone epiphyses of major limb bones of ungulates has shown in both BL and FN-3 an inverse relationship with their marrow weights, estimated as the mean for modern horse^34^ and bison^35^ (Fig. 2A). In contrast, the abundance of skeletal remains and bone portions correlates directly with their mineral densities, estimated as the mean of data from horse, wildebeest and raindeer^36^ (Fig. 2B).

### Cortical modifications

The analysis of bone surfaces allowed to identify biostratinomic modifications produced by hominins and carnivores. In addition, five bones showed gnaw marks made by rodents. Anthropic activity was evidenced by cut marks and percussion marks. Carnivore tooth marks included scores, pits, furrowing and crenulated edges^7^.

### Cut marks

Cut marks show similar frequencies in both assemblages. In the case of FN-3, the proportions are similar in the two archaeological levels, while in BL they concentrate in a single archaeological level, the D layer.

**Figure 3:**
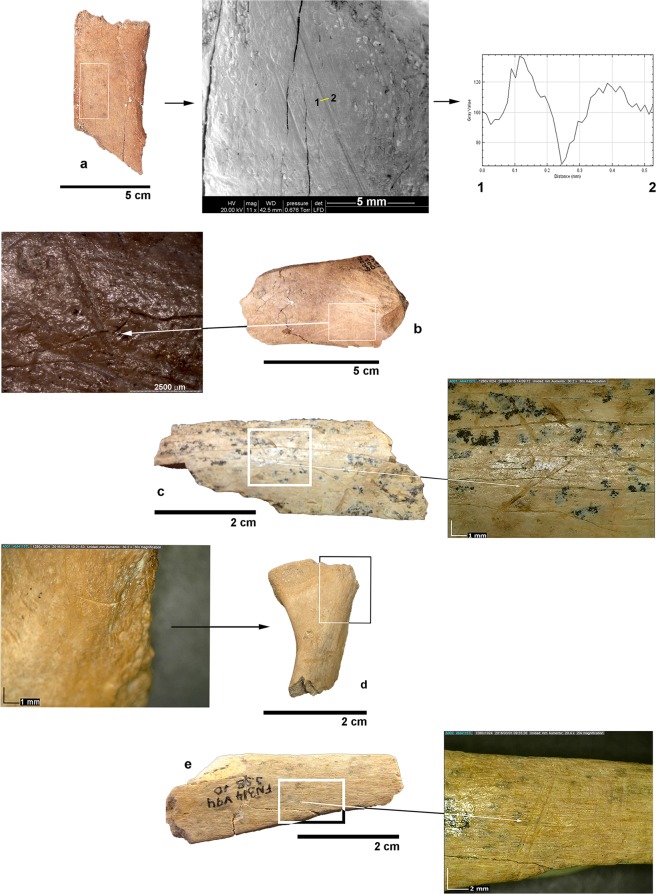
Examples of cut marks on the large mammal bones from BL and FN-3. Level D of BL. (**a**) incision on an external rib shaft fragment from a very large mammal, with a detail of its crop profile; (**b**) chop mark on a limb bone diaphyseal fragment of a medium-to-large sized mammal; FN-3. (**c**) incision on limb bone diaphyseal shaft of a large sized mammal; (**d**) incisions on a mandibular fragment of a small-to-medium sized mammal; (**e**) incision on an indeterminate bone fragment of a medium-to-large sized mammal.

Sixty-four bones showing cut marks were found in the bone samples analysed, 32 from each site (Table 1), which represent 0.75% of the remains analysed from BL and 0.83% in the case of FN-3. Most cut marks appear on skeletal remains from animals of medium-to-large and very large size (see details in Supplementary Tables S11 and S12). Given that the bones of small-sized species show a lower preservational completeness than those of large-sized ones, they are comparatively less represented in both assemblages. For this reason, most identifiable elements from the small size class correspond to craniodental remains (Supplementary Tables S3 and S4). This taphonomic bias implies that the intensity of anthropic action on the bones of small-sized taxa is probably underestimated in our study. Cut marks are relatively short (length range: 2.3–13.0 mm in BL, 1.8–10.1 mm in FN-3) and use to appear isolated or in pairs. Incisions are the principal type of modification in both sites, although scrapes, sawing marks, and chop marks are also documented. Depending on their type, cut marks appear on different anatomical portions, with a predominance of obliquely oriented incisions on the diaphyseal shafts of limb bones and the external face of ribs. Metapodials preserve cut marks in their middle and distal shaft, most of them showing a transverse orientation. Cut marks are scarcely represented on the cranial skeleton: there is only one mandibular fragment of the ascending ramus that shows a couple of transverse incisions situated near the condylar process (Fig. 3).

**Table 1:** Number of skeletal remains with cut marks and evidence of anthropic fractures in Level D from Barranco León (BL) and all fertile levels from Fuente Nueva-3 (FN-3).

	Cut-marked bones				Bones with anthropic fractures			
	BL		FN-3		BL		FN-3	
Skeletal element	n	%	n	%	n	%	n	%
Mandible			1	3.13				
Vertebra	2	6.25	1	3.13				
Rib	5	15.63	4	12.50				
Scapula	1	3.13			2	2.25		
Pelvis			1	3.13	1	1.12		
Humerus	2	6.25	1	3.13	2	2.25	1	1.35
Radius	1	3.13					1	1.35
Femur	1	3.13	1	3.13			2	2.70
Tibia	2	6.25			10	11.24	5	6.76
Unidentified limb bones	10	31.25	9	28.1	46	51.69	38	51.35
Metapodial	2	6.25	5	15.63	7	7.87	8	10.81
Calcaneum	1	3.13						
Phalanx					1	1.12	1	1.35
Unidentified elements	3	9.38	9	28.13	20	22.47	18	24.32
Carapace fragments	2	6.25						
TOTAL	32	100.00	32	100.00	89	100.00	74	100.00

See Supplementary Tables S11 and S12 for additional information on the cut marks (i.e., type, number of striations, location, orientation, butchery action involved, and size of the animal).

### Patterns of bone breakage

The faunal assemblages of BL and FN-3 preserve many fractured bones. Complete elements are scarce and correspond to isolated teeth, metapodials and carpal/tarsal bones. The autopodial elements are more mineralized than other bones of the appendicular skeleton and have a lower nutritional value, which makes them less attractive to hominins and carnivores^23,24^. Analysis of fracturation patterns^37^ evidences that fractures with an oblique angle and a curved, V-shaped outline predominate in both sites, followed by those showing right angles and longitudinal outlines. Smooth surfaces predominate in the fractures with curved and longitudinal angles, while jagged surfaces are more frequent in the transversal fractures (Supplementary Fig. S1). These features show the role of biostratinomic agents in the generation of both bone assemblages, as patterns of green bone breakage predominate over those on dry bone.

**Figure 4:**
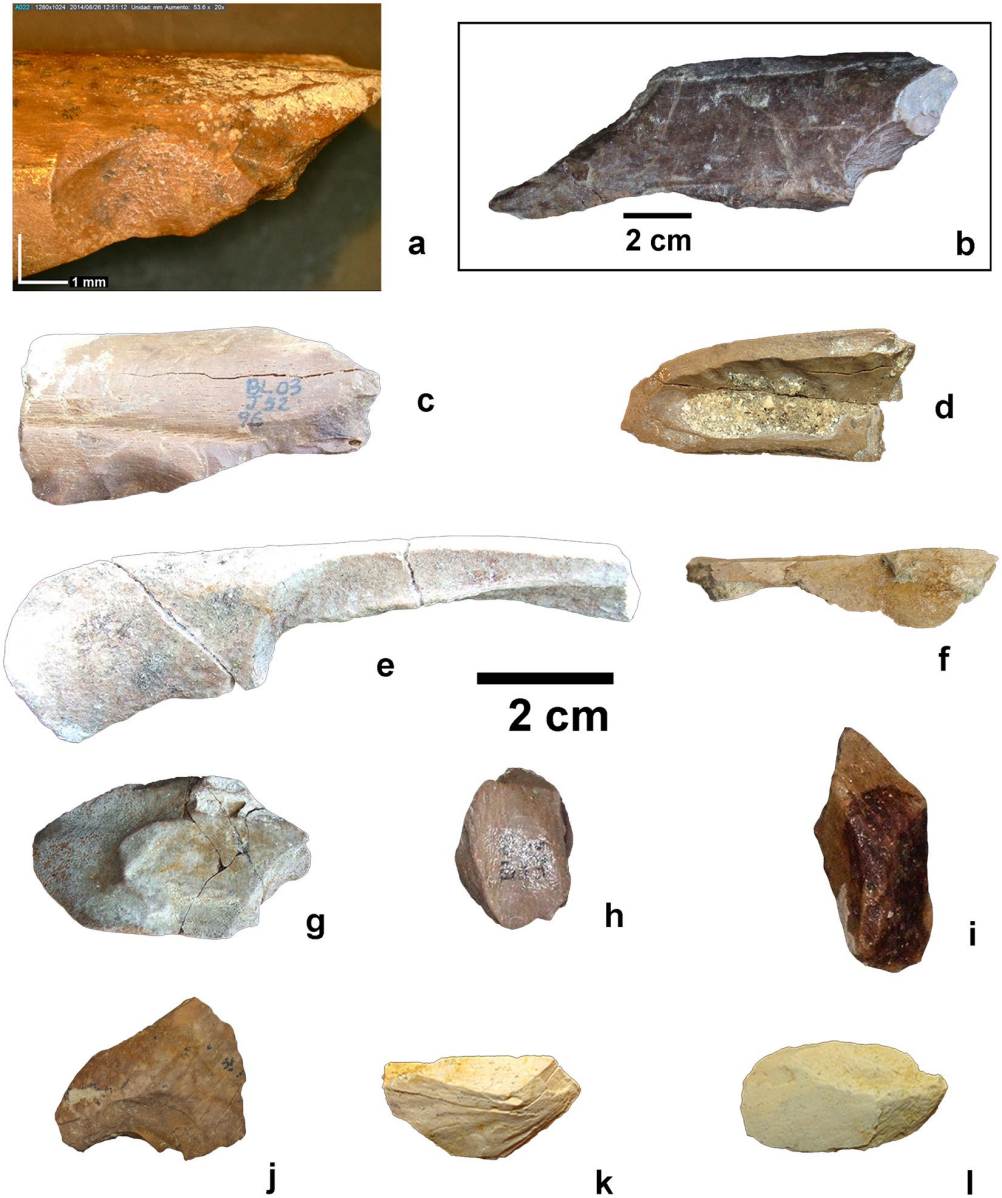
Examples of bones broken in fresh state from level D of BL and FN-3 levels. (**a**) diaphyseal fragment of a tibia from a small-to-medium sized mammal showing a conchoidal scar (BL-D); (**b**) limb bone diaphyseal fragment with a conchoidal scar (BL-D); (**c**) limb bone fragment of a medium-to-large sized mammal showing conchoidal scars (BL-D); (**d**) fractured humerus of a medium-to-small sized mammal (BL-D); (**e**) fractured limb bone (FN-3); f: limb bone fragment with percussion notches (FN-3); (**g**–i) impact flakes (BL-D); (**j**–**l**) impact flakes (FN-3).

Evidence of intentional bone fracturing by hominins was detected in 89 bones from level BL-D and 74 bones from all levels of FN-3 (Table 1), which represent 2.09% and 1.92% of the samples analysed from these sites, respectively, including percussion marks, pits, notches, impact flakes, and negative flake scars generated by direct contact of bones with the hammerstone in the impact (Fig. 4).

In both sites, evidence of bone fracturing by hominins concentrates on the major limb bones of large and medium-to-large sized mammals. Among the scarce remains that could be identified taxonomically, equids are the best recorded.

### Modification by carnivores

**Figure 5:**
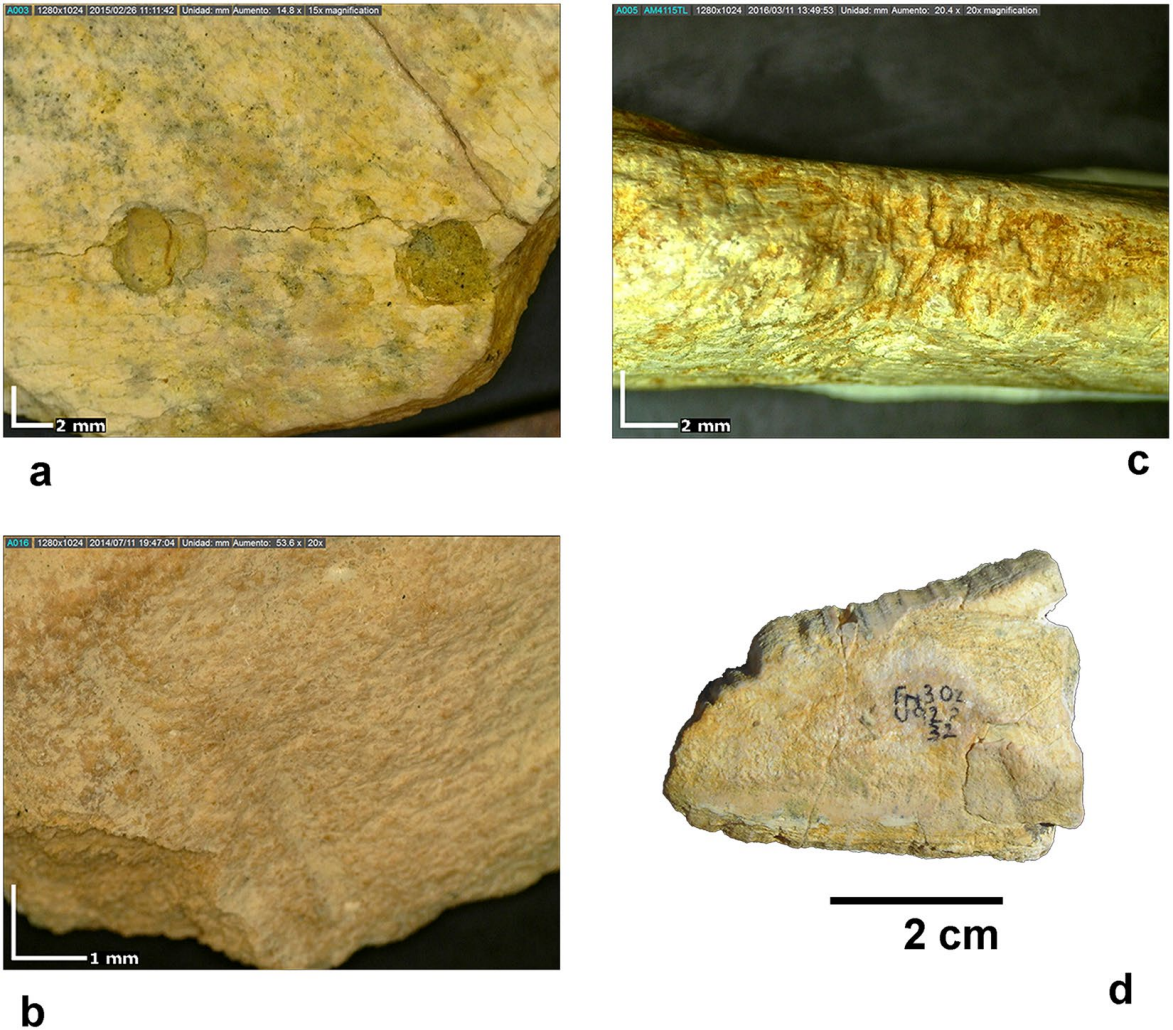
Examples of modifications by carnivores and rodents of large mammal bones from level D of BL and FN-3. (**a**) limb bone fragment of a large sized mammal from FN-3 showing 2 pits; (**b**) score on a flat bone fragment of a large sized mammal from BL-D; (**c**) second lateral metatarsal of *Equus altidens* from FN-3 showing scores produced by rodents; d: limb bone fragment tooth-marked by *Hystrix* sp.

Although hominins are the main agent responsible of bone fracturing, evidence of breakage by carnivores is also present at both sites, but in lower frequencies. Moreover, rodent activity is also evident in some elements, including a bone shaft with many gnawing marks of a porcupine at FN-3 (Fig. 5).

Carnivore damage of bones consists basically of isolated scores and pits, although furrowing, crenulated edges, pitting, and digested bones are also recognized (Fig. 5). Most tooth marks appear on stylopodial and zeugopodial bones, but some autopodial elements (mostly metapodials) show also tooth marks. With the exception of three rib fragments, the elements of the axial skeleton are no damaged by carnivores. By size classes, the bones most affected by carnivore activity belong to mammals of medium-to-large and large size.

### Other types of bone modifications

There are bones in both assemblages that show alterations by physico-chemical agents during the biostratinomic phase (e.g., weathering and dissolution in a humid environment) and also postdepositional modifications (e.g., iron and manganese mineralizations). In spite of this, the bone surfaces are generally well preserved, with most elements showing a low weathering stage^38^, except those that appeared close to the surface of the capping sediment, which outer surface evidences modification by plant roots of the modern vegetation. Moreover, rose diagrams show no evidence of preferred alignment of bones in FN-3 and BL, except for the lowermost part of level D, which preserves bones with polished edges due to hydraulic transport. In addition, the surface of the lithic tools is relatively fresh and all components of the operative lithic chain are present, including abundant debitage fragments of very small dimensions^29^.

## Discussion

Although the subsistence strategy of early *Homo* probably included a broad spectrum of dietary resources, “meat made us humans” is a recurrent topic in this issue^39,40^. Meat consumption by hominins is evidenced by the presence of bones with unequivocal cut marks and intentional bone breakage patterns in the faunal assemblages from BL and FN-3. This provides useful information on the behaviour of the first human populations that inhabited Western Europe. Moreover, the Oldowan lithic assemblages from both sites^29,30^ and the inferences on resource availability and competition intensity among the members of the carnivore guild^41^ make possible to discuss on the subsistence strategies of these human populations.

The lithic assemblages from BL and FN-3 are composed of abundant flakes of small size as well as cores and debris, largely made on flint and to a lesser extent on limestone^29,30^. The technological features of these lithic assemblages, including the small dimensions of the flint flakes (usually <2 cm), allow speculating on carcass acquisition by hominins, which relates to the classical debate on *Homo* as a “scavenger” or as a “hunter”^25,42–44^ (see supplementary discussion and references therein). The first scenario considers that early *Homo* with Oldowan tools would have secondary access to ungulate carcasses through passive scavenging of abandoned remains^18,42,45–47^. The second envisions hominins as having primary access to fully fleshed carcasses, with carcass acquisition resulting from hunting more likely than from power, confrontational scavenging or active searching^43,44,48,49^. However, it is difficult to conceive that the Orce hominins could have a direct impact on medium-to-large and very large ungulate prey using their small Oldowan flakes^50^.

In any case, although the scavenging of carcasses partially defleshed would have enhanced the survival of the hominin population of BL and FN-3^7,24,41,51,52^, this does not preclude other procurement strategies, including the opportunistic hunting of small-sized mammals, the kleptoparasitism of the prey of sabre-tooths like *Homotherium latidens* and *Megantereon whitei*, or even the scavenging of carcasses of very large animals that died from other causes. The latter possibility is documented in the Upper Level of FN-3, which preserves evidence of competition between *Homo* sp. and the giant, short-faced hyena *Pachycrocuta brevirostris* for the exploitation of the carcass of an old individual of *Mammuthus meridionalis*^7^. Moreover, the results of a model that allows estimating the biomass of primary consumers available in the community of large mammals suggest a scavenging behaviour as the optimal scenario for the hominin population that inhabited the depression of Baza and Guadix^41^.

Anthropic damage of bones is mainly documented in BL and FN-3 by the presence of cut marks. Incisions are the principal type of modification, while scrapes, sawing marks and chop marks are comparatively less abundant. The position of cut marks on the skeletal portions, as well as their morphology and type, relate with the anthropic activities performed in carcass processing. These in turn are largely conditioned by the anatomy and size of the animals. The oblique and longitudinal incisions in the midshafts of the limb bones relate with defleshing activities, while their presence in the proximal and distal epiphyses results from disarticulation. Scrape marks evidence periosteum removal. In the case of the metapodials and one calcaneum, sawing marks relate to their disarticulation, chop marks evidence the removal of tendons and incisions result from skinning and disarticulation. Incisions in the mandible indicate skinning and disarticulation, while in the pelvis and scapula result from defleshing. The incisions on the external face of ribs as well as on the apophyses and dorsal face of the vertebral bodies evidence defleshing. In contrast, those on the internal face of ribs and vertebral bodies indicate evisceration. The predominance of cut marks related to defleshing activities and evisceration of carcasses can be tentatively interpreted as indicating that the hominins had, at least from time to time, early access to ungulate carcasses that were nearly intact (Supplementary Tables S11 and S12).

The bones with evidence of anthropic breakage are more abundantly represented in both assemblages than those showing cut marks. Analysis of fracture patterns shows a systematic breakage of bones for marrow consumption, especially in the case of the major limb bones, although bone fracturing is present in other anatomical elements such as phalanges. This suggests that the exploitation of bone marrow after hammerstone breakage^30^ was a usual activity during carcass processing.

Analysis of the abundance of limb bone epiphyses and other skeletal portions in both sites has shown an inverse correlation with their estimated marrow yields and a positive one with their mineral densities (Fig. 2). This relationship is also documented at Venta Micena (VM), an Early Pleistocene site of Orce slightly older than BL and FN-3 that is interpreted as a denning area of *P*. *brevirostris*^23,24^. In the case of BL and FN-3, however, bone survival shows a tighter relationship with marrow weigth (Fig. 2A) than with mineral density (Fig. 2B), which is evidenced in a greater scatter around the regression line of the latter. In contrast, bone survival correlates with both factors to the same extent in VM. This probably results from the fact that hominins fractured the skeletal elements using their Oldowan tools, while hyenas used their enlarged premolar teeth, which forced them to be more selective in bone crushing.

Two plastron fragments of a tortoise from level BL-D preserve incisions and one of them shows also a scrape mark, probably related with the extraction of viscera. Apart from these cut-marked specimens, no evidence of consumption of other animal resources such as rodents, rabbits, birds (including eggs), reptiles and amphibians has been detected in the faunal assemblages from both sites. It is worth noting here that although the practice of a strategy based on small game is well documented in Middle and Late Pleistocene human communities, it is less frequently recorded during Early Pleistocene times^53–56^. Evidence of consumption of turtles is documented in a number of Middle and Late Pleistocene sites from Israel, Italy, Spain, and South Africa^54,57–59^. However, this evidence is scarce during the Early Pleistocene, being only recorded in East Turkana (Kenya) at 1.95 Ma^55^ and TE9 (Spain) at 1.2 Ma^59^. The two plastron fragments from BL-D with cut marks indicate that, at least occasionally, this resource was also exploited in the Early Pleistocene sites of Orce.

Most evidence on the subsistence strategies of hominins during Early Pleistocene times is based on cut marks recorded in carcasses of large mammals, although evidence on consumption of fish and reptiles is also available^60^. Similarly, consumption of fish and crabs is documented in the early Middle Pleistocene site of Gesher Benot Ya´aqov, dated to ~750 ka^61^.

From a taphonomic point of view, those strategies based on the exploitation of animal resources leave marks that can be preserved and recognized in the fossil record, but this does not apply to plant foods. For this reason, the evidence of butchery preserved in the fossil bones must be complemented with information compiled on the exploitation by extant hunters-gatherers of resources not usually preserved in the archaeopalaeontological sites. These studies have shown important differences in the percentage of food types included in their diet^15,16,20,62^. For example, plants are more relevant as dietary resources for many human populations than wild game, as in the case of the!Kung of the Kalahari Desert, whose diet comprises 60–80% of vegetable foods (e.g., nuts, fruits, berries, melons, roots, and bulbs) and consume also other animal foods apart from game (e.g., small mammals, birds, reptiles, eggs, insects, and honey). Similarly, the Hadza of Tanzania feed on a number of herbivorous mammals that range in size between impalas and elephants, and even hunt other carnivores or steal by power scavenging their prey, which represents 20% of those carcasses of medium-to-large size acquired. However, they rely mainly on edible vegetation and consume other foods such as insects and honey^16,20,62^.

Plant foods can be scarce or totally absent in some environments. An extreme situation is found among the Arctic peoples, who rely exclusively on fishing (69.99%) and hunting (30%) of sea and land mammals, including whales, walruses and caribou, which means that plant carbohydrates are nearly absent from their diet^63^. Lee^15^ argued on a positive relationship between the importance of animal resources and latitude: beyond 50° the primary source is hunting and, to a lesser extent, fishing, which relates to the decrease in the availability of edible plants. In contrast, gathering is the dominant mode of subsistence in latitudes similar to those of the Orce sites.

Unfortunately, all attempts to extract fossil palynomorphs from the sediments of the Orce sites have been unsuccessful. Coprolites are other source of pollen, but an analysis of several coprolites of *P*. *brevirostris* from these sites yielded negative results^64^.

In spite of the absence of fossil pollen, a combined approach based on ecomorphology and biogeochemistry of the fossil fauna of VM provided interesting clues on the palaeovegetation of the basin^65^. The predominance of taxa in this site adapted to a grazing diet supports the sinecological reconstruction of this palaeocommunity as an open plain with tree patches and bush/forest areas. Moreover, an estimate of 780 mm of annual palaeoprecipitations derived from the range of δ^15^N values in ungulates^22^ agrees with the rainfall calculated for BL-D and FN-3 from the herpetofauna^33,66^, 750 mm (these values are higher than the current annual rainfall in the basin, 300 mm on average).

Most ungulates of VM are present in BL-D and FN-3. The exceptions are the mesodont ovibovine *Soergelia minor*, which is replaced in FN-3 by the hypsodont caprine *Ammotragus europaeus*, and the presence of a second equid species in BL-D and FN-3, the large-sized horse *Equus sussenbornensis*^41^. Given that hindgut fermenters can feed on low quality grasses too fibrous for a ruminant to subsist on^65^ the record of two highly hypsodont, hypergrazing equids in BL and FN-3 suggests for these sites slightly drier conditions than in VM.

The lacustrine deposits that preserve the main archaeopalaeontological sites of the Baza basin represent a stratigraphic unit that shows an alternation of levels of marls-calcilutites and limestones, with intercalations of sands and conglomerates to the lake borders. This lake was fed by strontium- and sulphate-rich waters from hot springs originated from tectonic activity in the basin. As such, they represented ‘ecological islands’ of hot waters that would probably be advantageous for hominin settlements, as evidenced by a number of Plio-Pleistocene sites in the African Rift Valley found in similar palaeoenvironmental contexts^22^. Concerning the detritic facies, they are associated to organic-rich coastal lacustrine environments and emerged plains, which represented an ecosystem similar to that found in modern African savannahs with tree patches. The abundance in the lacustrine sediments of microfaunal remains, including euryhaline gastropods and ostracods that live in both freshwater and oligo- to mesosaline conditions^32^, indicates an organic-rich environment with brackish waters. This agrees with: (i) the presence of microcrystalline gypsum originated by neoformation^22^; (ii) the high δ^15^N values measured in the bone collagen of *Hippopotamus antiquus*, a species more dependent of the aquatic environment than the extant *H*. *amphibious*^22,65^; and (iii) the identification of skeletal remains of common shelduck (*Tadorna tadorna*) in VM, a waterfowl that today dwells in coastal mudflats, estuaries and riverine environments, feeding mostly on saltwater snail *Hydrobia*^67^. These hydrothermal environments with high plant productivity and standing crop biomass of ungulates were optimal for the first human settlements in Western Europe^22^.

Carnivoran modifications (tooth pits and scores) are present in both BL and FN-3. Insights on the size and type of the carnivores responsible of them can be derived from the analysis of their dimensions (length and breadth) as well as from the density and thickness of the bones in which they are preserved^68,69^. The large size of these tooth marks and their finding on bone shafts with a dense cortical surface suggest a large-sized carnivore as the bitting agent^69^. Moreover, the presence of notches on the bone edges resulting from bone crushing, a task not performed by felids, and the coincidence in morphology, dimensions and anatomical position of these tooth marks with those recorded at VM, a site where the activity of *P*. *brevirostris* is well documented^23^, suggests the involvement of this giant hyaenid. In addition, the abundance of coprolites, specially in the Upper Level of FN-3^7^, indicates the frequent presence of hyenas at this site. However, a low number of tooth marks from carnivores of small-to-medium size are also recorded, which suggests a minor role of some canid (probably *Canis mosbachensis*) in the modification of bones.

Bone modification by carnivores is much less frequent than that of anthropic origin. It is interesting the presence of three bones (one from BL and two from FN-3) that preserve cut marks and tooth marks, although there is no overlap between these marks. The scarcity of elements modified by both agents and the low frequency of tooth marks do not allow to make inferences on the pattern of interaction between hominins and carnivores at these sites. In any case, carnivore activity seems to have been residual compared to hominin activity, with the exception of the Upper Level of FN-3.

## Conclusions

Data on anthropic action from BL and FN-3 evidence that the subsistence strategy of the hominin populations that inhabited Europe in the Early Pleistocene involved the exploitation of carcasses of medium-to-large and large-to-very large sized animals for obtaining meat, fat and bone marrow. Other animals of small size available in the environment, including rodents, leporids, tortoises and birds, were also presumably consumed, together with eggs, honey and a wide spectrum of edible vegetation.

Carnivore activity is documented in a number of bones from BL and FN-3. Most tooth marks can be attributed to the giant hyena *P*. *brevirostris*. However, carnivore activity in these sites seems to have been residual compared to hominin activity.

## Materials and Methods

The palaeontological record unearthed from levels D1 and D2 of BL during systematic excavations and fieldwork between the years 1999 and 2015 is composed of 6,566 vertebrate remains (mostly large mammals) and coprolites. Other levels of the stratigraphic section of this site provided a comparatively low number of remains (129), which mainly correspond to non-identifiable bone shafts. In the case of FN-3, 9,041 fossils were recovered from all the archaeopalaeontological levels of the site during this period of time.

Anatomical and taxonomic data were recorded for most of these fossils (see refs. in Supplementary Table S1). Species of large mammals were distributed among size categories following refs^19,70^. These studies used seven size classes (i.e., 1 to 6, with a subdivision of class 3 in 3a and 3b), but we only considered the six main size classes (Table 2). Each element in which taxonomically diagnostic features were absent was classified to order, infraorder, family or tribu level, and then grouped in the appropriate size category.

**Table 2:** Size classes proposed by Brain^19^ and Bunn^70^, and their equivalences in this study (size classes 5 and 6 are grouped).

Class	kg	Size
1	<23	Small size (S)
2	23–114	Medium-to-small size (MS)
3a	114–227	Medium size (M)
3b	227–340	Medium-to-large size (ML)
4	340–907	Large size (L)
5	907–2,721	Very large size (VL)
6	>2,721	

The faunal assemblages of the sites were taphonomically analysed following the standard methodology^19,23,24,71,72^. Numbers of remains (NR), number of identified specimens (NISP), minimum numbers of elements (MNE), minimum numbers of individuals (MNI) and minimum anatomical units (MAU) were calculated for all taxa. Two age groups were established for the specimens, immature and adult individuals, with two subdivisions in each of them: immature individuals were grouped in newborns and juveniles, while adults were classified as adults sensu stricto (i.e., yearlings and prime adults) and past-prime adults (i.e., senile individuals). Criteria for estimating age at death included patterns of tooth replacement and degree of wearing for teeth (deciduous and permanent) as well as degree of epiphyseal fusion for the limb bones. Surface modification was only analysed in a part of the assemblage, because abundant bones are badly preserved and need restoration. For this reason, the sample analysed in search of modification traces in the cortical surface of bones is composed of 4,249 skeletal remains from BL-D and 3,852 from FN-3 levels (i.e., 64.7% and 42.6% of the fossil assemblages, respectively).

Bone cortical surfaces were analysed with a stereoscopic binocular microscope (Olympus SZ 11), a digital microscope (DINO-LITE Modell AM4115TL) and a SEM microscope (FEI QUANTA 600). Criteria used for identifying cut marks were based on refs^18,73–80^. This allowed to document the type of cut marks (i.e., incisions, sawing marks, scraping marks, and chop marks) and to record their anatomical location, orientation and number of striations. Criteria for identification of percussion marks were based on refs^78,81–84^. Cut marks were differentiated from those resulting from trampling using experimental data provided in ref.^80^. Percussion notches, pits, impact flakes, and negative flake scars were documented. Bone breakage patterns were classified according to ref.^37^. The crop profile of Fig. 3a was obtained using the software ImageJ v.1.50e (https://imagej.nih.gov/ij/).

Carnivoran activity was described based on refs^18,42,78,85,86^. Most tooth marks identified were pits and scores, although furrowing and crenulated edges were also present. Other bone surface modifications, including weathering, abrasion, root etching, and postdepositional alterations, were identified and described following refs^38,71,87,88^.

## Supplementary information

Supplementary information - The earliest cut marks of Europe: a discussion on hominin subsistence patterns in the Orce sites (Baza basin, SE Spain)

## Data Availability

All data used in this study are available in the supplementary tables and also on request from the corresponding author.

## References

[1] Carbonell E (2008). The first hominin of Europe. Nature.

[2] Cheheb RC (2019). Human behavior and Homo-mammal interactions at the first European peopling: new evidence from the Pirro Nord site (Apricena, Southern Italy). Sci. Nat..

[3] Michel V (2017). New dating evidence of the early presence of hominins in Southern Europe. Sci. Rep..

[4] Martínez-Navarro B, Turq A, Agustí J, Oms O (1997). Fuente Nueva-3 (Orce, Granada, Spain) and the first human occupation of Europe. J. Hum. Evol..

[5] Palmqvist P, Duval M, Diéguez A, Ros-Montoya S, Espigares MP (2016). On the fallacy of using orthogenetic models of rectilinear change in arvicolid teeth for stimating the age of the first human settlements in Western Europe. His. Biol..

[6] Duval M (2012). On the limits of using combined U-series/ESR method to date fossil teeth from two Early Pleistocene archaeological sites of the Orce area (Guadix-Baza basin, Spain). Quat. Res..

[7] Espigares MP (2013). Homo vs. Pachycrocuta: Earliest evidence of competition for an elephant carcass between scavengers at Fuente Nueva-3 (Orce, Spain). Quat. Int..

[8] Toro-Moyano I (2013). The oldest human fossil in Europe, from Orce (Spain). J. Hum. Evol..

[9] Semaw S (1997). 2.5-million-year-old stone tools from Gona, Ethiopia. Nature.

[10] de Heinzelin J (1999). Environment and behavior of 2.5-million-year-old Bouri hominids. Science.

[11] Sahnouni M (2018). 1.9-million-and 2.4-, million-year-old artifacats and stone tool-cutmarked bones from Ain Boucherit, Algeria. Science.

[12] Bunn, H. T. The bone assemblages from the excavated sites in Koobi Fora Research Project, Volume 5: Plio-Pleistocene Archaeology (ed. Isaac, G.) 402–458 (Clarendon Press, 1997).

[13] Bunn HT, Kroll EM (1986). Systematic butchery by Plio/Pleistocene hominids at Olduvai Gorge, Tanzania. Curr. Anthropol..

[14] Sahnouni M (2013). The first evidence of cut marks and usewear traces from the Plio-Pleistocene locality of El-Kherba (A n Hanech), Algeria: implications for early hominin subsistence activities circa 1.8 Ma. J. Hum. Evol..

[15] Lee, R. B. What hunters do for a living, or, how to make out on scarce resources in Man the hunter (eds Lee, R. B. & DeVore, I.) 30–48 (Aldine Publishing Co, 1968).

[16] Woodburn, J. An introduction to Hazda ecology in Man the hunter (eds Lee, R. B. & DeVore, I.) 49–55 (Aldine Publishing Co, 1968).

[17] Binford LR (1980). Willow smoke and dogs’tails: hunter-gatherer settlement systems and archaeological site formation. Am. Antiq..

[18] Binford, L. R. *Bones: Ancient Men and Modern Myths* (Academic Press, 1981).

[19] Brain, C. K. *The Hunters or the Hunted?**An Introduction to African Cave Taphonomy* (The University of Chicago Press, 1981).

[20] Hawkes K, O’connell JF, Blurton Jones G, Oftedal OT, Blumenshchine RJ (1991). Hadza: big game, common goods, foraging goals, and the evolution of the human diet. Phil. Trans. R. Soc. London.

[21] Marlowe FW (2005). Hunter-gatherers and human evolution. Evol. Anthr..

[22] García-Aguilar JM (2014). Hydrotermal activity and its paleoecological implications in the Latest Miocene to Middle Pleistocene lacustrine environments of the Baza Basin (Betic Cordillera, SE Spain). Quat. Sci. Rev..

[23] Arribas A, Palmqvist P (1998). Taphonomy and paleoecology of an assemblage of large mammals: hyaenid activity in the Lower Pleistocene site at Venta Micena (Orce, Guadix-Baza Basin, Granada, Spain). Geobios.

[24] Palmqvist P (2011). The giant hyena *Pachycrocuta brevirostris:* modelling the bone-cracking behavior of an extinct carnivore. Quat. Int..

[25] Martínez-Navarro B (2018). Oldowan scavengers vs. Acheulian hunters: what does the faunal record say? Glob. J. Arch. Anthropol..

[26] Álvarez C (2015). New magnetostratigraphic and numerical age of the Fuente Nueva-3 site (Guadix-Baza basin, Spain). Quat. Int..

[27] Martínez-Navarro B (2015). The Epivillafranchian and the arrival of pigs into. Europe. Quat. Int..

[28] Turq A (1996). Le Plio-Pléistocène dela région d’Orce, province de Grenada, Espagne: bilan et perspectives de Recherche. Paléo.

[29] Toro-Moyano I (2011). The archaic stone tool industry from Barranco León and Fuente Nueva 3 (Orce, Spain): Evidence of the earliest hominin presence in southern. Europe. Quat. Int..

[30] Titton S (2018). Active percussion tools from the Oldowan site of Barranco León (Orce, Andalusia, Spain): the fundamental role of punding activities in hominin lifeways. J. Archaeol. Sci..

[31] Oms O, Anadón P, Julià R (2011). Geology and chronology of the continental Pleistocene archeological and paleontological sites of the Orce area (Baza basin, Spain). Quat. Int..

[32] Anadón P, Gabàs M (2009). Paleoenvironmental evolution of the Early Pleistocene lacustrine sequence at Barranco León archeological site (Orce, Baza basin, Southern Spain) from stable isotopes and Sr and Mg chemistry of ostracod shells. J. Paleolimnol..

[33] Blain HA (2016). Refining upon the climatic background of the Early Pleistocene hominid settlement in Western Europe: Barranco León and Fuente Nueva-3 (Guadix-Baza Basin, SE Spain). Quat. Sci. Rev..

[34] Outram A, Rowley-Conwy P (1988). Meat and marrow utility indices for horse (*Equus*). J. Archaeol. Sci..

[35] Brink JW (1997). Fat content in leg bones of *Bison bison*, and applications to archaeoloy. J. Archaeol. Sci..

[36] Lam YM, Chen X, Pearson OM (1999). Intertaxonomic variability in patterns of bone density and the differential representation of bovid, cervid, and equid elements in the archaeological record. Am. Antiq..

[37] Villa P, Mahieu E (1991). Brakage patterns of human long bones. J. Hum. Evol..

[38] Behrensmeyer AK (1978). Taphonomic and ecologic information from bone weathering. Paleobiology.

[39] Stanford, C. B. *Meat Eating and the Origins of Human**Behaviour* (Princeton University Press,1999).

[40] Bunn, H.T. Meat made us human in Evolution of the Human Diet (ed. Ungar, P.) 191–211 (Oxford University Press, 2007).

[41] Rodríguez-Gómez G (2016). On the ecological context of the earliest human settlements in Europe: resource availability and competition intensity in the carnivore guild of Barranco León-D and Fuente Nueva-3 (Orce, Baza Basin, SE Spain). Quat. Sci. Rev..

[42] Blumenschine RJ (1995). Percussion marks, tooth marks, and experimental determination of the timing of hominid and carnivore access to long bones at FLK Zinjanthropus, Olduvai Gorge, Tanzania. J. Hum. Evol..

[43] Domínguez-Rodrigo, M., Egeland, C. P. & Barba, R. The hunting-versus scavenging debate in Deconstructing Olduvai: a Taphonomic Study of the Bed I Sites (eds Domínguez-Rodrigo, M., Barba, R. & Egeland, C. P.) 11–22 (Springer, 2007).

[44] Domínguez-Rodrigo M, Bunn HT, Yravedra J (2014). A critical re-evaluation of bone surface modification models for inferring fossil hominin and carnivore interactions through a multivariate approach: application to the FLK Zinj archaeofaunal assemblage (Olduvai Gorge, Tanzania). Quat. Int..

[45] Blumenschine, R. J. *Early Hominid Scavenging Opportunities: Implications of Carcass Availability in the Serengeti and Ngorongoro Ecosystems* (B.A.R. Int. Ser. 283, Oxford, 1986).

[46] Capaldo SD (1997). Experimental determinations of carcass processing by Plio-Pleistocene hominids and carnivores at FLK 22 (Zinjanthropus), Olduvai Gorge, Tanzania. J. Hum. Evol..

[47] Selvaggio MM (1998). Evidence for a three-stage sequence of hominid and carnivore involvement with long bones at FLK Zinjanthropus, Olduvai Gorge, Tanzania. J. Archaeol. Sci..

[48] Domínguez-Rodrigo M, Barba R (2006). New estimates of tooth mark and percussion mark frequencies at the FLK Zinj site: the carnivore-hominid-carnivore hypothesis falsified. J. Hum. Evol..

[49] Bunn HT, Pickering TR (2010). Bovid mortality profiles in paleoecological context falsify hypotheses of endurance running-hunting and passive scavenging by early Pleistocene hominins. Quat. Res..

[50] Blumenschine, R. J. & Pobiner, B. L. *Zooarchaeology and the ecology of Oldowan hominin carnivory in Evolution of the Human Diet* (ed. Ungar, P.) 167–190 (Oxford University Press, 2007).

[51] Martínez-Navarro, B. Early Pleistocene faunas of Eurasia and hominid dispersals in The First Hominin Colonization of Eurasia, Contributions from the Second Stony Brook Human Evolution Symposium and Workshop (eds Fleagle, J. G., Shea, J. J., Grine, F. E., Baden, A. L. & Leakey, R. E.) 207–224 (Springer, 2010).

[52] Palmqvist P, Torregrosa V, Pérez-Claros JA, Martínez-Navarro B, Turner A (2007). A re-evaluation of the diversity of *Megantereon* (Mammalia, Carnivora, Machairodontinae) and the problem of species identification in extinct carnivores. J. Vert. Paleontol..

[53] Steadman DW, Plourde A, Burley DV (2002). Prehistoric butchery and consumption of birds in the Kingdom of Tonga, South Pacific. J. Archaeol. Sci..

[54] Blasco R (2008). Human consumption of tortoises at Level IV of Bolomor Cave (Valencia, Spain). J. Archaeol. Sci..

[55] Braun DR (2010). Early hominin diet included diverse terrestrial and aquatic animals, 1,95 Ma in East Turkana, Kenya. Proc. Nat. Acad. Sci. USA.

[56] Rufá A, Blasco R, Rivals F, Rosell J (2014). Leporids as a potential resource for predator (hominin, mammalian, carnivores, raptors): an example of mixed contribution from Level III of Teixoneres Cave (MIS 3, Barcelona, Spain). C.R. Palevol..

[57] Blasco R (2016). Tortoises as a dietary supplement: a view from the Middle Pleistocene site of Qesem Cave, Israel. Quat. Sci. Rev..

[58] Biton R, Gonen S, Oron M, Steiner T, Rabinovich R (2017). Freshwater turtle or tortoise? The exploitation of testudines at the Mousterian site of Nahal Mahanayeem Outlet, Hula Valley, Israel. J. Archaeol. Sci. Rep..

[59] Blasco R (2011). Earliest evidence for human consumption of tortoises in the European Early Pleistocene from Sima del Elefante, Sierra de Atapuerca, Spain. J. Hum. Evol..

[60] Archer W, Braun DR, Harris JWK, McCoy JT, Richmon BG (2014). Early Pleistocene aquatic resource use in the Turkana Basin. J. Hum. Evol..

[61] Alperson-Afil N (2009). Spatial organization of hominin cativities at Gesher Benot Ya’aqov, Israel. Science.

[62] Bunn, H. T. Hunting, power scavenging, and butchering by Hadza foragers and by Plio-Pleistocene Homo in Meat-Eating and Human Evolution (eds Stanford, C. B. & Bunn, H. T.) 199–218 (Oxford University Press, 2001).

[63] Binford, L. R. *Constructing Frames of Reference. An Analytical Method for Archaeological Theory Building Using Ethnographic and Environmental Data Sets* (University of California Press, 2001).

[64] Carrion JS (2009). Quaternay pollen analysis in the Iberian Peninsula: the value of negative results. Internet Archaeol..

[65] Palmqvist P, Pérez-Claros JA, Gröcke DR, Janis CM (2008). Tracing the ecophysiology of ungulates and predator-prey relationships in an early Pleistocene large mammal community. Palaeogeogr. Palaeoclimatol. Palaeoecol..

[66] Blain HA, Bailón S, Agusti J, Martínez-Navarro B, Toro I (2011). Paleoenvironmental and paleoclimatic proxies to the Early Pleistocene hominids of Barranco León D and Fuente Nueva 3 (Granada, Spain) by means of their amphibian and reptile assemblages. Quat. Int..

[67] Carboneras, C. & Kirwan, G. M. Common shelduck (*Tadorna tadorna*) in Handbook of the Birds of the World Alive (eds del Hoyo, J., Elliott, A., Sargatal, J., Christie, D. A., de Juana, E.) (Lynx Edicions, Barcelona, 2018).

[68] Delaney-Rivera C (2009). Pits and pitfalls: taxonomic variability and patterning in tooth marks dimensions. J. Archaeol. Sci..

[69] Andrés M, Gidna AO, Yravedra J, Domínguez-Rodrigo M (2012). A study of dimensional differences of tooth marks (pits and scores) on bones modified by small and large carnivores. Archaeol. Anthropol. Sci..

[70] Bunn, H. T. *Meat-Eating and Human Evolution: Studies on the Diet and Subsistence Patterns of Plio-Pleistocene Hominids in East Africa* (Ph. D. dissertation. University of California, Berkeley 1982).

[71] Lyman, R. L. *Vertebrate Taphonomy* (Cambridge University Press, 1994).

[72] Reitz, E. & Wing, E. S. *Zooarchaeology* (Cambridge University Press,1999).

[73] Bunn HT (1981). Archaeological evidence for meat-eating by Plio-Pleistocene hominids from Koobi Fora and Olduvai Gorge. Nature.

[74] Shipman, P. Applications of scannning electron microscopy to taphonomic problems in The Research Potential of Anthropological Museum Collections (eds Cantwell, A. M., Griffin, J. B. & Rosthschild, N. A.). Ann. New York Acad. Sci. **376**, 357–385 (1981). 10.1111/j.1749-6632.1981.tb28179.x 7041753

[75] Pots R, Shipman P (1981). Cutmarks made by stone tools on bones from Olduvai Gorge, Tanzania. Nature.

[76] Bromage TG, Boyde A (1984). Microscopic criteria for the determination of directionality cutmarks on bone. Am. J. Phys. Anthrop..

[77] Noe-Nygaard N (1989). Man-made trace fossils on bones. Hum. Evol..

[78] Blumenshchine RJ, Marean CW, Capaldo SD (1996). Blind test of interanalyst correspondence and accuracy in the identification of cut marks, percussion marks, and carnivore tooth marks on bone surfaces. J. Archaeol. Sci..

[79] Blumenschine RJ, Prassack KA, Kreger CD, Pante MC (2007). Carnivore tooth marks, microbial bioerosion, and the invalidation of Domínguez-Rodrígo and Barba’s (2006) test of Oldowan hominin scavenging behavior. J. Hum. Evol..

[80] Domínguez-Rodrigo M, De Juan S, Galán AB (2009). A new protocol to differentiate trampling marks from butchery cut marks. J. Archaeol. Sci..

[81] Blumenschine RJ, Selvaggio MM (1988). Percussion marks on bone surfaces as a new diagnostic of hominid behavior. Nature.

[82] Capaldo SD, Blumenschine RJ (1994). A quantitative diagnosis of notches made by hammerstone percussion and carnivore gnawing on bovid long bones. Am. Antiq..

[83] Pickering TR, Egeland CP (2006). Experimental patterns of hammerstone percussion damage on bones and zooarchaeological inferences of carcass processing intensity by humans. J. Archaeol. Sci..

[84] Galán AB, de Juana S, Domínguez-Rodrigo M (2009). A new experimental study on percussion marks and notches and their bearing on the interpretation of hammerstone-broken faunal assemblages. J. Archaeol. Sci..

[85] Haynes G (1980). Evidence of carnivore gnawing on Pleistocene and recent mammalian bones. Paleobiology.

[86] Shipman, P. Early hominids lifestyle: hunting and gathering or foraging and scavenging in Animals and Archaeology, vol 1: Hunters and their Prey (eds Clutton-Brock, J. & Grigson, C.) 31–49 (BAR 163,1983).

[87] Fiorillo AR (1988). Taphonomy of Hazard Homestead Quarry (Ogallala Group), Hitchcock County, Nebraska. Vols. Contributions to Geology, University of Wyoming.

[88] Fernández-Jalvo Y, Andrews P (2003). Experimental effects of water abrasion on bone fragments. J. Taphon..

