# Supplementary material for: The earliest cut marks of Europe: a discussion on hominin subsistence patterns in the Orce sites (Baza basin, SE Spain)
Source: Sci Rep. 2019 Oct 28;9:15408. doi: 10.1038/s41598-019-51957-5 (PMC6817892; doi:10.1038/s41598-019-51957-5)
Supplement: Supplementary file 1 — Supplementary information - The earliest cut marks of Europe: a discussion on hominin subsistence patterns in the Orce sites (Baza basin, SE Spain) [file 41598_2019_51957_MOESM1_ESM.pdf]

Supplementary information

The earliest cut marks of Europe: a discussion on hominin subsistence patterns in the Orce sites (Baza basin, SE Spain)

M. Patrocinio Espigares[1*], Paul Palmqvist[1], Antonio Guerra-Merchán[1], Sergio Ros-Montoya[1], José Manuel García-Aguilar[1], Guillermo Rodríguez-Gómez[1], Francisco J. Serrano[1], Bienvenido Martínez-Navarro[2,3,4]

[1] Departamento de Ecología y Geología, Universidad de Málaga, Campus de Teatinos, 29071 Malaga, Spain ; [2] IPHES, Institut Català de Paleoecologia Humana i Evolució Social, C/ Marcel.lí Domingo s/n, Campus Sescelades, Edifici W3, 43007 Tarragona, Spain ;[3] Area de Prehistoria, Universitat Rovira i Virgili (URV), Avda. Catalunya 35, 43002 Tarragona, Spain; [4] ICREA, Pg. Lluís Companys 23, 08010 Barcelona, Spain .

* Corresponding author

Supplementary Tables and Figures

Supplementary Table S1. Faunal assemblages from Barranco Léon (BL) and Fuente Nueva-3 (FN-3).

Genus & Species	BL	FN-3	Reference
Aves indet.	x	x	Espigares, 2010
Discoglossus cf. D. *jeanneae*	x	x	Blain et al., 2016
Pelobates cultripes	x		Blain et al., 2016
Bufo bufo	x		Blain et al., 2016
Bufo calamita	x		Blain et al., 2016
Bufo sp.	x	x	Blain et al., 2016
Hyla meridionalis	x		Blain et al., 2016
Rana cf. R. *perezi*	x	x	Blain et al., 2016
Anura indet.	x	x	Blain et al., 2016
Chalcides cf. *bedriagae*		x	Blain et al., 2016
cf. *Chalcides*	x		Blain et al., 2016
Lacerta cf. *lepida*	x	x	Blain et al., 2016
Lacertidae	x	x	Blain et al., 2016
Ophisaurus sp.	x		Blain et al., 2016
cf. *Ophisaurus*		x	Blain et al., 2016
Natrix maura	x	x	Blain et al., 2016
Natrix natrix	x	x	Blain et al., 2016
Rhinechis scalaris	x	x	Blain et al., 2016
Malpolon monspessulanus	x	x	Blain et al., 2016
Colubridae	x	x	Blain et al., 2016
Ophidien indet.	x		Blain et al., 2016
Emys cf. E. orbicularis	x		Blain et al., 2016
Mauremys cf. M. leprosa	x		Blain et al., 2016
Testudo sp.	x	x	Blain et al., 2016
Asoriculus gibberodon	x	x	Furió, 2010
Sorex minutus	x	x	Furió, 2010
Sorex sp.	x	x	Furió, 2010
Crocidura sp.	x	x	Furió, 2010
Erinaceus cf. praeglaciaris	x	x	Furió, 2010
Galemys sp.	x	x	Furió, 2010
Mimomys savini	x	x	Agustí et al., 2010
Allophaiomys cf. *lavocati*	x	x	Agustí et al., 2010
Allophaiomys sp.	x	x	Agustí et al., 2010
Castillomys rivas	x	x	Agustí et al., 2010
Apodemus flavicollis	x		Agustí et al., 2010
Apodemus mystacinus		x	Agustí et al., 2010

Oryctolagus cf. *lacosti*.	x	x	Agustí et al., 2010
Prolagus sp.	x		Agustí et al., 2010
Hystrix sp.	x	x	Martínez Navarro et al., 1997
Lynx cf. pardinus		x	Boscaini et al., 2015
Pachycrocuta brevirostris	x	x	Martínez Navarro et al., 2010
Lycaon lycaonoides	x	x	Martínez Navarro et al., 2010
Canis mosbachensis	x	x	Martínez Navarro et al., 2010
Vulpes cf. *praeglacialis*	x	x	Martínez Navarro et al., 2010
Ursus etruscus	x	x	Medin et al., 2017
Pannonictis cf. *nestii*	x	x	Martínez Navarro et al., 2010
Meles meles	x	x	Madurell-Malapeira et al., 2011
Mammuthus meridionalis	x	x	Ros-Montoya et al.,, 2010
Stephanorhinus hundsheimensis	x	x	Lacombat, 2010
Equus altidens	x	x	Alberdi, 2010
Equus sussenbornensis	x	x	Alberdi, 2010
Hippopotamus antiquus	x	x	Martínez Navarro et al., 2010
Bison sp.	x	x	Martínez Navarro et al., 2010
Hemitragus albus	x	x	Martínez Navarro et al., 2010
Ammotragus europaeus		x	Moullé et al., 2004
Praemegaceros cf. *verticornis*	x	x	Abazzi, 2010
Metacervocerus rhenanus	x	x	Abazzi, 2010

Supplementary Table S2: Numbers of identifiable elements (NISP) of carnivores and ungulates, and numbers of remains (NR) of indeterminate mammals from Level D of Barranco León (BL) and all levels of Fuente Nueva-3 (FN-3). Data for microfauna, herpetofauna, aves and coprolites not included.

	BL	FN3		
		Lower level	Upper Level	Indet. Level
Carnivores	50	21	131	9
Ungulates	1,437	557	1,244	507
Mammal indet.	4,718	1,474	4,671	279

Supplementary Table S3: Number of identifiable elements (NISP) and minimum number of elements (MNE) by anatomical parts and size classes in the faunal assemblage of Level D of Barranco León (BL).

BL				Very large size		Large size		Medium-to-large size		Medium size		Medium-to-small size		Small size		Unident. size
**Anatomical elements**	**NR TOTAL**	**NISP TOTAL**	**MNE TOTAL**	**NISP**	**MNE**	**NISP**	**MNE**	**NISP**	**MNE**	**NISP**	**MNE**	**NISP**	**MNE**	**NISP**	**MNE**	**NR**
Horn	6	6	3			1	1	1	1			2	1			2
Antler	20	20	5			4	1					7	4			9
Skull	43	43	10	8	3	8	3	5	1	4	1	2	1	2	1	14
Maxilla	6	6	3	1	1	1	1			1	1					3
Mandible	33	33	12	3	2	13	5	3	1	3	1	5	3			6
Isolated teeth	1,762	1,762		346		640		77		147		113		15		424
Vertebra	86	86	18	10	4	18	6	7	2	8	1	6	2	7	3	30
Rib	99	99	13	6	2	36	2	13	3	14	2	11	2	5	2	14
Scapula	17	17	6	2	1	3	2	2	1			2	1	2	1	6
Pelvis	32	32	8	1	1	8	2	3	1	3	1	3	2	2	1	12
Humerus	39	39	17	4	3	14	7			2	1	7	4	6	2	6
Radius	18	18	6			10	3	1	1	3	1			2	1	2
Ulna	7	7	4	1	1	4	1			1	1			1	1	
Femur	25	25	9	4	1	8	3			2	1	1	1	5	3	5
Tibia	39	39	10	2	1	21	6			4	1	6	1	1	1	5
Fibula	1	1	1			1	1									
Carpals	28	28	24	4	3	14	14	0	0	0	0	7	6	1	1	2
Patella	3	3	3	2	2							1	1			
Tarsals	82	82	59	1	1	49	37	0	0	1	1	18	14	6	6	7
Carpals/tarsals	23	23		4		6		1		3				2		7
Malleolus	4	4	3			1	1					1	1	1	1	1
Metacarpal	31	31	9	1	1	18	8	0	0	0	0	0	0	0	0	9
Metatarsal	32	32	16	1	1	17	12	0	0	1	1	9	2	0	0	4
Metapodial	63	63	24	2	2	29	11	5	0	2	1	13	9	6	1	6
Sesamoideum	10	10	7	1	1	7	4	1	1	1	1					
Phalanx	72	72	47	3	3	39	28	0	0	2	2	16	8	7	6	5
Limb bones	805			10		327		151		196		51		34		36
Flat bones	184			3		56		31		56		19		9		10
Carapace fragment	306													306		
Unidentified element	2,659															
Coprolites	31															
**Total**	**6,566**	**2,581**	**317**	**420**	**34**	**1,353**	**159**	**301**	**12**	**454**	**18**	**300**	**63**	**420**	**31**	**625**

Supplementary Table S4: Number of identifiable elements (NISP) and minimum number of elements (MNE) by anatomical parts and size categories from each archaeological level of FN-3.

FN-3 Lower/Upper/Indet Level				Very large size		Large size		Medium-to-large size		Medium size		Medium-to-small size		Small size		Unident. size
ELEMENTO	NR TOTAL	NISP TOTAL	MNE TOTAL	NISP	MNE	NISP	MNE	NISP	MNE	NISP	MNE	NISP	MNE	NISP	MNE	NR
Horn	1/8/-	1/8/-	1/2/-	-/-/-	-/-/-	1/1/-	1/1/-	-/-/-	-/-/-	-/-/-	-/-/-	-/1/-	-/1/-	-/-/-	-/-/-	-/6-
Antler	18/15/11	18/15/11	9/2/7	-/-/-	-/-/-	2/3/2	1/1/1	-/-/-	-/-/-	-/-/-	-/-/-	9/2/6	8/1/4	-/-/-	-/-/-	7/10/3
Skull	22/66/5	22/66/5	5/10/2	2/10/1	2/3/1	7/21/-	2/2/-	-/4/-	-/1/-	-/2/-	-/1/-	2/3/-	1/1/-	-/3/-	-/2/-	11/23/4
Maxilla	3/8/-	3/8/-	1/6/-	-/1/-	-/1/-	1/4/-	1/3/-	-/-/-	-/-/-	-/-/-	-/-/-	-/1/-	-/1/-	-/1/-	-/1/-	2/1/-
Mandible	18/53/7	18/53/7	6/23/6	4/14/1	2/8/1	4/16/4	2/7/3	-/1/-	-/1/-	-/2/-	-/1/-	1/1/1	1/1/1	1/6/1	1/5/1	8/15/-
Isolated teeth	626/1427/427	626/1427/427		68/474/113		337/355/274		3/14/-		4/12/2		43/40/17		7/13/1		164/519/20
Vertebra	52/103/9	52/103/9	10/18/1	4/10/-	1/3/-	10/34/3	5/11/1	-/6/-	-/1/-	5/4/-	2/1/-	1/2/-	1/1/-	-/2/-	-/1/-	32/49/6
Rib	44/227/2	44/227/2	6/12/1	6/28/-	1/3/-	10/34/-	1/3/-	1/4/-	1/1/-	3/26/-	1/2/-	1/4/-	1/1/-	4/2/-	1/2/-	19/129/2
Scapula	11/20/4	11/20/4	1/5/2	1/2/-	1/1/-	-/3/1	-/2/1	-/-/-	-/-/-	-/1/-	-/1/-	-/1/-	-/1/-	-/-/1	-/-/1	10/13/2
Pelvis	18/24/4	18/24/4	1/5/2	-/2/-	-/1/-	2/6/2	1/1/2	-/2/-	-/1/-	-/1/-	-/1/-	-/2/-	-/1/-	-/-/-	-/-/-	16/11/2
Humerus	12/37/8	12/37/8	6/12/6	2/6/2	1/3/1	2/12/6	1/4/5	-/2/-	-/1/-	1/1/-	1/1/-	2/-/-	2/-/-	1/5/-	1/3/-	4/11/-
Radius	17/11/6	17/11/6	6/4/3	3/1/1	2/1/1	3/5/2	2/2/1	-/-/-	-/-/-	2/-/-	1/-/-	1/1/1	1/1/1	-/-/-	-/-/-	8/4/2
Ulna	3/9/1	3/9/1	2/3/1	1/-/1	1/-/1	1/6/-	1/3/-	-/-/-	-/-/-	-/-/-	-/-/-	-/-/-	-/-/-	-/-/-	-/-/-	1/3/-
Femur	8/18/2	8/18/2	5/4/1	1/2/1	1/1/1	4/5/-	3/2/-	-/-/-	-/-/-	-/2/-	-/1/-	2/-/-	1/-/-	-/-/-	-/-/-	1/9/1
Tibia	9/43/9	9/43/9	3/9/5	-/2/4	-/1/1	8/25/3	3/6/3	-/-/-	-/-/-	-/3/1	-/1/1	-/-/-	-/-/-	-/1/-	-/1/-	1/12/1
Fibula	1/1/-	1/1/-	1/1/-	-/-/-	-/-/-	-/-/-	-/-/-	-/-/-	-/-/-	-/-/-	-/-/-	-/-/-	-/-/-	-/-/-	-/-/-	1/1/-
Carpals	4/16/7	4/16/7	4/13/6	1/5/2	1/5/1	3/7/5	3/7/5	-/-/-	-/-/-	-/1/-	-/1/-	-/-/-	-/-/-	-/-/-	-/-/-	-/3/-
Patella	4/2/2	4/2/2	3/1/1	-/1/-	-/1/-	2/-/1	2/-/1	-/-/-	-/-/-	-/-/-	-/-/-	1/-/-	1/-/-	-/-/-	-/-/-	1/1/1
Tarsals	17/37/14	17/37/14	14/25/14	6/5/-	6/5/-	7/21/11	2/17/11	-/-/-	-/-/-	-/-/-	-/-/-	4/-/3	4/-/3	2/3/-	2/3/-	2/8/-
Carpals/tarsals	9/34/5	9/34/5		3/8/-		4/11/2		-/3/-		-/-/-	-/-/-	1/-/2		-/-/-	-/-/-	2/2/1
Malleolus	-/-/5	-/-/5		-/-/-	-/-/-	-/-/5	-/-/5	-/-/-	-/-/-	-/-/-	-/-/-	-/-/-	-/-/-	-/-/-	-/-/-	-/-/-

Metacarpal	10/9/14	10/9/14	8/5/11	-/-/-	-/-/-	9/6/14	7/4/11	-/-/-	-/-/-	-/-/-	-/-/-	1/1/-	1/1/-	-/-/-	-/-/-	/2/-
Metatarsal	17/20/10	17/20/10	6/13/9	-/2/-	-/2/-	13/17/10	5/10/9	-/-/-	-/-/-	-/-/-	-/-/-	3/1/-	1/1/-	-/-/-	-/-/-	1/-/-
Metapodial	30/47/8	30/47/8	6/4/5	-/-/-	-/-/-	18/29/3	5/4/3	-/-/-	-/-/-	-/-/1	-/-/1	2/-/1	1/-/1	-/-/-	-/-/-	9/-/3
Sesamoideum	4/15/2	4/15/2	3/12/1	-/1/-	-/1/-	3/10/1	3/9/1	-/-/-	-/-/-	-/-/-	-/-/-	-/-/-	-/-/-	-/2/-	-/2/-	1/2/1
Phalanx	22/59/14	22/59/14	18/38/12	-/4/-	-/4/-	11/31/7	11/27/7	-/1/-	-/1/-	-/-/-	-/-/-	3/4/6	3/4/5	2/2/-	2/2/-	4/17/1
Limb bones	160/569/7			9/23/-		39/122/1		12/11/-		18/40/3		2/12/-		3/15/1		77/179/2
Flat bones	40/122/1			7/12/-		13/43/-		-/5/-		13/21/-		1/-/-		2/2/-		4/39/1
Carapace fragment	19/38/3			-/-/-		-/-/-		-/-/-		-/-/-		-/-/-		19/38/3		-/-/-
Unidentified element	965/2831/264			12/29/1		27/141/7		27/33/-		4/47/-		4/20/-		15/14/-		896/2703/256
Coprolites	1/156/0															
**Total**	**2,165/6,025/851**	**980/2,309/576**	**125/230/96**	**130/613/127**	**19/44/8**	**531/968/364**	**62/116/70**	**43/86/-**	**1/6/-**	**50/163/7**	**5/11/2**	**84/96/37**	**27/16/15**	**56/89/7**	**7/21/2**	**1,282/3,772/309**

Supplementary Table S5: Number of remains (NR), number of identifiable elements (NISP) and minimum number of elements (MNE) by taxa in the faunal assemblage of Barranco León (BL). As most elements in the size categories are bone fragments that cannot be attributed to a given anatomical element, the size categories include only the number of remains (NR).

**BL**	Survey			B Level			C Level			D level			E Level			F Level			Indet. Level		
**Taxa**	NR	NISP	MNE	NR	NISP	MNE	NR	NISP	MNE	NR	NISP	MNE	NR	NISP	MNE	NR	NISP	MNE	NR	NISP	MNE
Megantereon whitei										1	1	1									
Lynx cf. *pardinus*										1	1	1									
Pachycrocuta brevirostris										2	2	2									
Lycaon lycaonoides										1	1	1									
Canis mosbachensis										6	6	6									
Vulpes cf. *praeglacialis*										6	6	6									
Ursus etruscus										8	8	6									
Meles meles										3	3	3									
Pannonictis sp.										2	2	2									
Mammuthusmeridionalis										11	11	2									
Stephanorhinus hunsheimensis										61	61	12	1	1	1						
Equus altidens										32	32	32							3	3	3
Equus sussenbornensis							3	3	3	16	16	16	1	1	1						
Equus altidens/sussenbornensis	1	1	1	2	2	2	11	11	10	471	471	306	2	2	2	1	1	1	2	2	2
Hippopotamus antiquus	1	1	1				5	5	1	302	302	52	2	2	2				1	1	1
Bison										58	58	52	2	2	2						
Hemitragus albus										30	30	26									
Ammotragus europaeus										1	1	1									
Praemegaceros cf. *verticornis*										93	93	61	1	1	1						
Metacervoceros rhenanus										111	111	85	4	4	4						

Hystrix sp.										1											
Oryctolagus cf. lacosti										8	6	6									
Ave indet.										5	2	2									
Chelonia indet.										316			5								
Very large size										15											
Large size										365			1								
Medium-to-large size										115			1								
Medium size							1			207			2								
Medium-to-small size										85			4								
Small size										77			2								
Unidentified element	2						1			4,156			63			4					
Total	4	2	2	2	2	2	21	19	14	6,566	1,216	673	91	13	13	5	1	1	6	6	6

Supplementary Table S6: Minimum number of individuals (MNI) by age classes in the faunal assemblage of Barranco León (BL).

BL	**MNI (immature/adult)**						
Taxa/Level	Survey	B Level	C Level	D Level	E Level	F Level	Indet.
Megantereon whitei				0/1			
Lynx cf. *pardinus*				0/1			
Pachycrocuta brevirostris				0/1			
Lycaon lycaonoides				0/1			
Canis mosbachensis				0/1			
Vulpes cf. *praeglacialis*				0/2			
Ursus etruscus				0/1			
Meles meles				0/1			
Pannonictis sp.				0/1			
Mammuthusmeridionalis				0/1			
Stephanorhinus hunsheimensis				0/2	0/1		
Equus sp.	0/1	0/1	0/1	4/9	1/1	1/0	
Equus altidens				1/2			0/1
Equus sussenbornensis			0/1	0/2	0/1		
Hippopotamus antiquus	0/1		0/1	2/3	0/1		
Bison sp.				1/3	0/1		
Hemitragus albus				0/3			
Ammotragus europaeus				0/1			
Praemegaceros cf. *verticornis*				1/4	0/1		
Metacervoceros rhenanus				2/6	0/1		

Supplementary Table S7: Number of remains (NR), number of identifiable elements (NISP) and minimum number of elements (MNE) by taxa in the FN-3 assemblage. As most elements in the size categories are bone fragments that cannot be attributed to a given anatomical element, the size categories include only the number of remains (NR).

**FN-3**	Lower Level			Upper Level			Indet. Level		
**Taxa**	NR	NISP	MNE	NR	NISP	MNE	NR	NISP	MNE
Homotherium latidens							1	1	1
Lynx cf. *pardinus*				2	2	2			
Pachycrocruta brevirostris	3	3	2	7	7	7			
Lycaon lycaonoides	1	1	1	3	3	3	1	1	1
Canis mosbachensis	5	5	4	10	10	9			
Vulpes cf. *praeglacialis*	2	2	2	7	7	6			
Ursus etruscus	3	3	3	25	25	22	4	4	4
Meles meles				3	3	3			
Pannonictis cf. *nestii*				2	2	2	1	1	1
Mammuthus meridionalis	66	66	14	344	344	61	60	60	12
Stephanorhinus hundsheimensis	13	13	8	108	108	37	30	30	9
Equus altidens	321	321	246	100	75	75	232	232	192
Equus sussenbornensis	17	17	16	4	4	4	13	13	13
Equus altidens/sussenbornensis	37	37	27	170	170	107	4	4	4
Hippopotamus antiquus	24	24	14	144	144	58	27	27	9
Bison sp.	27	26	26	51	51	37	64	64	60
Hemibos sp. cf. *gracilis*	1	1	1	1	1	1			
Hemitragus albus	14	14	13	19	19	17	10	10	10
Ammotragus europaeus							2	2	2
Praemegaceros cf. *verticornis*	23	23	20	87	87	57	35	35	28
Metacervoceros rhenanus	46	46	38	17	17	16	26	26	20
Oryctolagus cf. *lacosti*	3	3	3	9	6	6	0		
Ave indet.	2	1	1	4	2	2	1	1	1
Chelonia indet.	18			36			3		
Very large size	14			51			11		
Large size	110			382			16		
Medium-to-large size	21			77			1		
Medium size	44			134			7		
Medium-to-small size	6			28			0		
Small size	18			28			1		
Unidentified element	1,235			4,086			301		
Total	2,074	606	439	5,939	1,087	532	871	511	367

Supplementary Table S8: Minimum number of individuals (MNI) by age categories in the FN-3 assemblage.

FN-3	MNI (immature/Adult)		
Taxa/Level	Lower	Upper	Indet.
Homotherium latidens	-	-	0/1
Lynx cf. *pardinus*	-	0/1	-
Pachycrocruta brevirostris	0/1	0/2	-
Lycaon lycaonoides	0/1	0/1	0/1
Canis mosbachensis	0/1	0/1	-
Vulpes cf. *praeglacialis*	0/1	0/2	-
Ursus etruscus	0/1	0/2	0/1
Meles meles	-	0/1	-
Pannonictis cf. *nestii*	0/0	0/2	0/1
Mammuthus meridionalis	2/0	6/2	-
Stephanorhinus hundsheimensis	0/1	2/3	0/1
Equus altidens	5/9	1/3	0/10
Equus sussenbornensis	0/2	1/1	0/2
Equus altidens/sussenbornensis	1/2	2/5	0/1
Hippopotamus antiquus	0/1	2/2	0/1
Bison sp.	0/3	1/5	1/0
Hemibos sp. cf. *gracilis*	0/1	0/1	-
Hemitragus albus	1/3	0/3	0/2
Ammotragus europaeus	-	-	0/1
Praemegaceros cf. *verticornis*	0/1	1/3	0/2
Metacervoceros rhenanus	2/2	1/2	1/1

Suplementary Table S9: %Minimal Anatomic Units (MAU) according to size categories in the faunal assemblage of Level D from Barranco León (BL). Teeth isolated from crania or mandibles are not included.

Anatomical element	Very large size	Large size	Medium-to-large size	Medium size	Medium-to-small Size	Small size
Skull	59.41	59.41	19.80	19.80	59.41	19.80
Maxilla	9.90	9.90		9.90		
Mandible	19.80	49.50	9.90	9.90	29.70	
Vertebra	2.18	0.63				
Rib	0.71					
Pelvis	9.90	19.80	9.90	9.90	19.80	9.90
Scapula	9.90	19.80	9.90		9.90	9.90
Humerus	29.70	69.31		9.90	39.60	19.80
Radius		29.70	9.90	9.90		9.90
Ulna	9.90	9.90		9.90		9.90
Femur	9.90	29.70		9.90	9.90	29.70
Tibia	9.90	59.41		9.90	9.90	9.90
Fibula		9.90				
Patella	19.80				9.90	
Malleolum		9.90			9.90	9.90
Carpals		22.83			16.50	
Tarsals	1.41	55.84	1.98		29.70	3.96
Metacarpal	2.48	59.41				
Metatarsal	2.48	59.41			19.80	
Metapodial	2.44	44.55			40.59	1.19
Sesamoideum						
Lateral metapodial		34.65				
Phalanx	4.06	100.00		2.48	17.33	2.51

Supplementary Table S10: %Minimal Anatomic Units (MAU) according to size categories and archaeological levels (Lower/Upper/Indet. Level) in the FN-3 faunal assemblage. Teeth isolated from crania/mandibles and the partial skeleton of *M. meridionalis* are not included.

Anatomical element	Very large size	Large size	Medium-to-large size	Medium size	Medium-to-small size	Small size
Skull	50/60/21.3	75/40/21.3	-/20/-	-/20/-	100/40/42.6	-/40/-
Maxilla	-/10/-	12.5/30/-	-/-/-	-/-/-	-/10/-	-/10/-
Mandible	25/80/10.6	25/70/31.9	-/10/-	-/10/-	12.5/10/10.6	12.5/50/10.6
Vertebra	0.9/2.2/-	57.8/16.6/0.7	-/0.7/-	1.9/0.8/-	1/0.8/-	-/0.8/-
Rib	0.3/1.4/-	0.7/1.7/-	1/0.6/-	1/1.5/-	1/0.8/-	1/2/-
Pelvis	-/10/-	12.5/10/21.3	-/10/-	-/10/-	-/10/-	-/-/-
Scapula	12.5/10/-	-/20/10.6	-/-/-	-/10/-	-/10/-	-/-/-
Humerus	12.5/30/10.6	12.5/60/53.2	-/10/-	12.5/10/-	25/-/-	12.5/10/-
Radius	25/10/10.6	25/20/10.6	-/-/-	12.5/-/-	12.5/10/10.6	-/-/-
Ulna	12.5/-/10.6	12.5/30/-	-/-/-	-/-/-	-/-/-	-/-/-
Femur	12.5/10/10.6	37.5/20/-	-/-/-	-/10/-		
Tibia	-/10/10.6	37.5/60/31.9	-/-/-	-/10/-	-/-/-	-/10/-
Fíbula						
Patella	-/-/-	25/-/10.6	-/-/-	-/-/-	12.5/-/-	-/10/-
Malleolum	-/-/-	-/-/53.2	-/-/-	-/-/-	-/-/-	-/-/-
Carpals	1.8/6.8/1.6	5.6/10.4/8.7	-/-/-	-/-/-	-/-/-	-/-/-
Tarsals	3.9/7.6/-	13/37/23.3	-/-/-	-/-/-	10/-/6.7	-/6/-
Metacarpal	-/-/-	100/40/100	-/-/0.2	-/-/-	12.5/10/-	-/-/-
Metatarsal	-/10.6/-	62.5/100/95.7	-/-/-	-/-/-	12.5/10/-	-/-/-
Metapodial	-/-/-	18.8/11.6/16	-/-/-	-/-/-	6.3/-/5.3	-/-/-
Sesamoideum	2.3/0.5/-	9.4/18.8/-	-/-/-	-/-/-	-/-/-	-/0.5/-
Lat. metapodial	-/-/-	43.8/45/-	-/-/-	-/-/-	-/-/-	-/-/-
Phalanx	-/2.9/32	48.9/83/-	-/5/-	-/-/-	5.1/10/11.5	0.2/0.3/-

Supplementary Table S11: Cut marks distributed among taxa and skeletal element in Level D of Barranco León (BL). Obl: oblique, Long: longitudinal, Trans: transversal, VL: very large size, L: large size, ML: medium-to-large size, MS: medium-to-small-size, I: indet. size.

Skeletal element	Taxon/ size category	Number of cut-marked bones	Number of striations	Type	Location	Orientation	Butchering action
Rib	VL	1	1	Incision	Rib dorsal shaft	Obl	Defleshing
	L	1	1	Incision	Internal margin	Obl	Defleshing
	L	1	1	Incision	Rib dorsal shaft	Obl	Defleshing
	L	1	4	Incision	Rib dorsal Shaft	Long	Defleshing
	ML	1	2	Incision	Rib dorsal shaft	Obl	Defleshing
Vertebra	L	1	2	Incision	Vertebral Body left lateral	Obl	Defleshing
	ML	1	1	Incision	Vertebral Body ventral	Obl	Evisceration
Scapula	ML	1	2	Incision	Proximal fragment.	Obl	Defleshing
Humerus	*Hippopotamus antiquus*	1	1	Incision	Diaphysis	Obl	Defleshing
	L	1	1	Incision	Diaphysis	Trans	Defleshing
Radius	ML	1	1	Incision	Prox. Epiphysis	Obl	Disarticulation
Femur	L	1	3	Incision	Prox. Epiphysis	Obl	Disarticulation
Tibia	L	2	1	Incision	Diaphysis	Obl	Defleshing
Limb bones	L	6	1	Incision	Diaphysis	Obl	Defleshing
	L	1	2	Incision	Diaphysis	Obl	Defleshing
	L	1	2	Incison	Diaphysis	Long	Defleshing
	ML	1	2	Incision-Chop mark	Diaphysis	Obl	Defleshing
	MS	1	1	Incision	Diaphysis	Obl	Defleshing
Metacarpal	L	1	3	Incision-Saw mark	Diaphysis	Long-Trans	Disarticulation
Metapodial	L	1	4	Incision	Diaphysis	Obl	Disarticulation
Calcaneum	L	1	3	Saw mark	Dorsal face	Trans	Disarticulation
Indet. bone	L	2	2	Incision			
	I	1	1	Incision			
Carapace fragment	Chelonia indet.	1	1	Incision	Plastron frag.		Evisceration
	Chelonia indet.	1	2	Incision-Scrapes	Plastron frag.		Evisceration

Supplementary Table S12: Cut marks distributed among taxa and skeletal elements from FN-3. Obl: oblique, Long: longitudinal, Trans: transversal, VL: very large size, L: large size, ML: medium-to-large size, MS: medium-to-small-size, I: indet. size.

Skeletal element	Taxon/ size category	Number of cut-marked bones	Number of striations	Type	Location	Orientation	Butchering action
**LOWER LEVEL**							
Mandible	MS	1	2	Incision	Vertical ramus	Obl-Trans	Skinning-disarticulation
Rib	L	1	2	Incision	Rib dorsal shaft	Long-Trans	Defleshing
Humerus	L	1	1	Incision	Diaphysis	Obl	Defleshing
Femur	*Equus*	1	1	Incision	Diaphysis	Obl	Defleshing
Limb bones	L	1	1	Incision	Diaphysis	Obl	Defleshing
	L	1	2	Incision	Diaphysis	Obl	Defleshing
	L	1	4	Incision	Diaphysis	Obl	Defleshing
	L	1	1	Scrapes	Diaphysis	Obl	Periostium removal
	ML	2	1	Incision	Diaphysis	Obl	Defleshing
Metatarsus	*Equus*	1	1	Chop mark	Metaphysis	Obl	Disarticulation-Tendon removal
Metapodial	L	1	1	Incision	Diaphysis	Trans	Skinning-Disarticulation
	L	1	2	Incision	Diaphysis	Trans	Skinning-Disarticulation
Indet. bones	I	1	2	Incision			
	I	1	3	Incision			
**UPPER LEVEL**							
Rib	ML	1	6 (3 groups)	Incision	Rib ventral shaft	Obl	Evisceration
	M	1	2	Incision	Rib dorsal shaft	Obl	Defleshing
	M	1	1	Incision	Rib dorsal shaft	Trans	Defleshing
Vertebra	L	1	3	Incision	Dorsal spine	Trans	Defleshing
Pelvis	L	1	1	Incision	Acetabulum	Trans	Disarticulation
Limb bones	L	1	1	Incision	Diaphysis	Obl	Defleshing
	ML	1	2	Incision	Diaphysis	Obl	Defleshing
	M	1	2	Incision	Diaphysis	Obl	Defleshing
Metacarpal	*Praemegaceros*	1	2	Incision	Diaphysis	Trans	Skinning-Disarticulation
Indet. bones	VL	1	2	Incision			
	ML	2	2	Incision			
	ML	1	1	Incision			
	M	1	3	Incision			
	I	2	1	Incision			
**INDET. LEVEL**							
Metacarpal	*Equus*	1	1	Incision	Diaphysis	Obl	Disarticulation

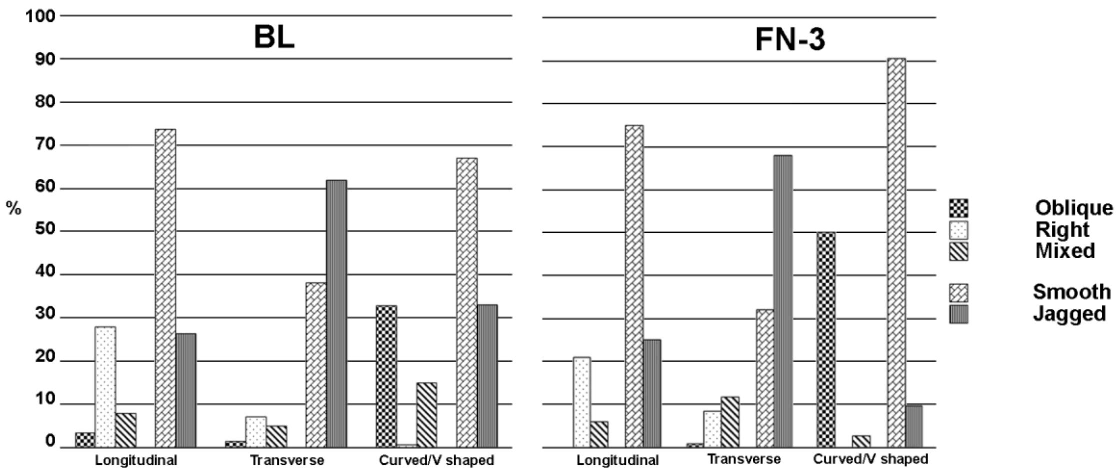

Supplementary Fig. S1: Distribution of bone breakage patterns in the bone assemblages from level D of BL and all levels of FN-3, including the relative abundances of fracture outlines (longitudinal, transverse, curved/V-shaped), fracture angles (right, oblique, mixed) and fracture edges (smooth, jagged).

References cited in Supplementary Table S1

Abazzi, L. La fauna de cérvidos de Barranco León y Fuente Nueva 3 in *Ocupaciones Humanas en el Pleistoceno inferior y medio de la cuenca de Guadix-Baza, Memoria Científica* (eds. Toro, I., Martínez-Navarro, B.& Agustí, J.) 273-290 (Junta de Andalucía. Consejería de Cultura. E.P.G. Arqueología Monográfico, 2010.

Agustí, J., de Marfá, R. & Santos Cubedo, A. Roedores y lagomorfos (Mammalia) del Pleistoceno inferior de Barranco León y Fuente Nueva 3 (Orce, Granada) in *Ocupaciones Humanas en el Pleistoceno inferior y medio de la cuenca de Guadix-Baza, Memoria Científica* (eds. Toro, I., Martínez-Navarro, B.& Agustí, J.) 121-140 (Junta de Andalucía. Consejería de Cultura. E.P.G. Arqueología Monográfico, 2010.

Alberdi, M.T. Estudio de los caballos de los yacimientos de Fuente Nueva-3 y Barranco León-5 (Granada) in *Ocupaciones Humanas en el Pleistoceno inferior y medio de la cuenca de Guadix-Baza, Memoria Científica* (eds. Toro, I., Martínez-Navarro, B.& Agustí, J.) 291-306 (Junta de Andalucía. Consejería de Cultura. E.P.G. Arqueología Monográfico, 2010.

Blain, H.A. *et al*. Refining upon the climatic background of the Early Pleistocene hominid settlement in Western Europe: Barranco León and Fuente Nueva-3 (Guadix-Baza Basin, SE Spain). *Quat. Sci. Rev.* **144,** 132-144 (2016).

Boscaini, A., Madurell-Malapeira, J., Llenas, M. & Martínez-Navarro, B. The origin of the critically endangered Iberian lynx: Speciation, diet and adaptive changes. *Quat. Sci. Rev.* **123,** 247-253 (2015).

Espigares, M.P. *Análisis y modelización del contexto sedimentario y los atributos tafonómicos de los yacimientos pleistocénicos del borde nororiental de la cuenca de Guadix-Baza* (Ph. D. Dissertation. University of Granada, Spain, 2010).

Furió, M. Contribución al conocimiento de los insectívoros (Insectivora,

Mammalia) del Pleistoceno inferior de Barranco León y Fuente Nueva 3 (Orce, Granada) in *Ocupaciones Humanas en el Pleistoceno inferior y medio de la cuenca de Guadix-Baza, Memoria Científica* (eds. Toro, I., Martínez-Navarro, B.& Agustí, J.) 141-164 (Junta de Andalucía. Consejería de Cultura. E.P.G. Arqueología Monográfico, 2010.

Lacombat, F. Estudio Paleontológico de *Stephanorhinus hundsheimensis* de Fuente Nueva 3 y Barranco León in *Ocupaciones Humanas en el Pleistoceno inferior y medio de la cuenca de Guadix-Baza, Memoria Científica* (eds. Toro, I., Martínez-Navarro, B.& Agustí, J.) 237-246 (Junta de Andalucía. Consejería de Cultura. E.P.G. Arqueología Monográfico, 2010.

Madurell-Malapeira, J. *et al.* The earliest European badger (*Meles meles*), from the Late Villafranchian site of Fuente Nueva 3 (Orce, Granada, SE Iberian Peninsula). *C. R. Palevol* **10,** 609-615 (2011).

Martínez-Navarro, B. *et al.* La fauna de grandes mamíferos de Fuente Nueva-3 y Barranco León-5: Estado de la cuestión in *Ocupaciones Humanas en el Pleistoceno inferior y medio de la cuenca de Guadix-Baza, Memoria Científica* (eds. Toro, I., Martínez-Navarro, B.& Agustí, J.) 197-236 (Junta de Andalucía. Consejería de Cultura. E.P.G. Arqueología Monográfico, 2010.

Martínez-Navarro, B., Turq, A., Agustí, J. & Oms, O. Fuente Nueva-3 (Orce, Granada, Spain) and the first human occupation of Europe. *J. Hum. Evol.* **33,** 611-620 (1997).

Medin T. *et al.* Late Villafranchian *Ursus etruscus* and other large carnivorans from the Orce sites (Guadix-Baza basin, Andalusia, southern Spain): Taxonomy, biochronology, paleobiology, and ecogeographical context. *Quat. Int.* **431,** 20-41 (2017).

Moulle, P.-E., Echassoux, A. & Martinez-Navarro, B.,. *Ammotragus europaeus*: une nouvelle espece de Caprini (Bovidae, Mammalia) du Pleistocene inferieur a la grotte du Vallonnet (France). *C. R. Palevol* **3,** 663-673 (2004).

Ros-Montoya, S., Palombo, M.R., Espigares, M.P. & Martínez-Navarro, B., 2010. La Sucesión de Proboscídeos en el Plio-Pleistoceno de las Cuencas de Guadix-Baza y de Granada (España) in *Ocupaciones Humanas en el Pleistoceno inferior y medio de la cuenca de Guadix-Baza, Memoria Científica* (eds. Toro, I., Martínez-Navarro, B.& Agustí, J.) 247-272 (Junta de Andalucía. Consejería de Cultura. E.P.G. Arqueología Monográfico, 2010.

Supplementary Discussion S1

Although the diet of early *Homo* probably included a broad spectrum of food resources, "meat made us humans" is a recurrent topic in any debate on the evolution of the human genus (Stanford, 1999; Bunn, 2007; Hardy 2010; Bunn et al., 2017; Hardy et al. 2017; Prado-Nóvoa et al., 2017). This debate is largely based on the evolutionary trend documented in the human fossil record to increasing encephalization, which runs in parallel to a trend to decreasing the size of the post-canine teeth (Jiménez-Arenas et al., 2012, 2014). The latter trend reflects the progressive adaptation of hominins to a higher-quality, easier-to-digest diet, which resulted in a shortening of the gastrointestinal tract. This forced the members of *Homo* to a greater consumption of meat compared with the australopithecines (Aiello and Wheeler, 1995; Bunn, 2001; Dunsworth and Walker, 2002).

Humans differ from other anthropoids in being the most genuine omnivorous species of the order Primates. As such, we show a number of anatomical and physiological adaptations to a more carnivorous diet than that of the great apes, our closest living relatives, and these adaptations probably arose in Africa at the origin of the genus *Homo* ~2.6 Myrs ago. For example, the gastrointestinal tract of *H. sapiens* is 40% shorter than the one expected for a primate of our size (Chivers and Hladik, 1980; Henneberg et al., 1998). As a result, the ratio of intestinal length to body length of humans (5:1) is lower than the one of baboons (8:1), not to speak on those of truly herbivorous mammals like the horse (12:1) and cattle (20:1), but is included within the range of values depicted by the extant carnivores (6-4:1). Similarly, our ratio of gastrointestinal surface area to body surface area (0.8:1) is close to the value of carnivores (0.6:1) and lower than those of baboon (1.1:1), horse (2.2:1) and cattle (3:1). Moreover, the coefficient of gut differentiation (i.e., surface area of stomach, caecum and colon divided by surface area of the small intestine) shows a value in humans (0.8) that is intermediate between those of carnivores (0.6-0.4), on the one hand, and the frugivorous chimpanzee (1.2) and orang (1.04), on the other, and that is half the value shown by the herbivorous gorilla (1.6) (Chivers and Hladik, 1980; Milton, 2003). In fact, the small intestine and colon represent in humans 67% and 17% of the total volume of the gastrointestinal tract, respectively. In contrast, this proportion is reversed in the great apes, ranging between 14-28% and 52-54%, respectively (Mann, 2000). The consequence of having a shorter colon than those of other primates is that

humans have a shorter food-passage time, which decreases the absorption of fibre-rich vegetal foods (Milton and Demment, 1988). Moreover, the enterocytes of the human digestive tract absorb with preference the iron linked to the haemoglobin and the porphyrin compounds, while the assimilation of ionic iron from plants is reduced by 50-70% due to the presence of phytates and phenolic compounds (Cook, 1990; Pereira and Vicente, 2013). For this reason, a vegan diet does not satisfy the human daily demands of iron (1.5 mg per day) and must be supplemented.

Dietary quality relates inversely in primates with body mass and with metabolic rate per unit mass, which allows the great apes to subsist with a diet in which vegetal matter represents between 87% and 99% (Milton, 2003). However, the human diet is more digestible and more energy-rich (in kJ per day and kg of body mass) than the one expected from our metabolic rate, which is the typical for a primate of our body mass (Leonard and Robertson, 1996; Henneberg et al., 1998). This relates to the high maintenance costs of the nervous tissue, which sums up to 20-25% of the basal metabolic rate in humans compared with 8-10% in chimpanzees (Leonard and Robertson, 1994).

According to the "expensive-tissue hypothesis" (Aiello and Wheeler, 1995), brain expansion competed with other tissues that have high maintenance costs, such as the heart, kidneys and splanchnic organs (liver and gastrointestinal tract). In this way, the trend to increasing encephalization in the genus *Homo* required to allocate to the nervous system a greater fraction of the energy budget invested in body maintenance. This required to cut the metabolic expenditure of other tissues with high maintenance costs. The size of the human heart could not be reduced, because a biped stance requires more heart pressure than a quadrupedal one to supply the brain with blood. Similarly, any reduction in either kidney size or energetic expenditure would have reduced the maximum concentration of urine excreted. A more dilute urine would in turn have been problematic for early *Homo* in the open habitats, where drinking opportunities are scarce and thermoregulatory requirements place considerable demands on the water budget (Wheeler, 1991). In the case of the liver, the size of this organ is constrained by the particular energy requirements of the brain, which uses glucose exclusively as its fuel. For these reasons, given that the gut is the only metabolically expensive organ that could be reduced in size, the expansion of the human brain forced a shortening of the gastrointestinal tract compared to the dimensions expected for the digestive system in a great ape of our size, and this

caused a shift to a more carnivorous diet compared to the australopithecines (Aiello and Wheeler, 1995). Moreover, a number of taeniid tapeworms that in their adult stage are characteristic parasites in carnivorous mammals use *Homo sapiens* as a definitive host. These parasites belong to the *Taenia solium* and *T. saginata-T. asiatica* subclades, and probably represent the result of two independent host shifts to hominins in sub-Saharan Africa. This suggests that an omnivorous diet, dependent on scavenging bovid prey taken by large felids and hyaenids, provided the ecological context for the evolution of *Taenia* specialized in early *Homo* as a definitive host (Hoberg et al., 2001).

Meat consumption by early *Homo* is evidenced by anthropic activity on bones of large mammals in a huge number of Pleistocene localities. In such sites, the finding of abundant cut marks, percussion marks and bone fracturing patterns evidence the importance of meat and bone marrow for these populations.

In the case of the sites studied here, Barranco León (BL) and Fuente Nueva-3 (FN-3), many bones of their faunal assemblages preserve unequivocal cut marks and evidence of intentional bone breakage. This contributes information on the dietary behaviour of the first human settlers of Western Europe. As discussed in the main text, the tool assemblages from both sites (Barsky et al., 2010; Toro-Moyano et al., 2011; Titton et al., 2018) are composed of abundant flakes of small size, cores and debris, largely made on flint and, to a lesser extent, on limestone. Moreover, the technological features of these assemblages, including the small dimensions of the flakes (usually <2 cm), and the inferences on resource availability and competition intensity among the members of the carnivore guild in the Early Pleistocene community of large mammals (Rodríguez-Gómez et al., 2016, 2017) make possible to discuss on the subsistence strategies of these human populations, including carcass acquisition. This relates to the classical debate on *Homo* as a “hunter” or as a “scavenger” (for review and references, see Blumenshine, 1995; Blumenshine et al., 2007; Domínguez-Rodrigo et al., 2007, 2014; Espigares et al., 2013; Martínez-Navarro et al., 2014; Rodríguez-Gómez et al., 2016; Martínez-Navarro, 2018).

Since the early eighties, two competing hypotheses on carcass acquisition by hominins have been subject to debate. A number of authors (e.g., Binford, 1981, 1985; Blumenschine, 1986, 1987, 1991, 1995; Blumenschine et al., 1994; Capaldo, 1997; Selvaggio, 1998; Arribas and Palmqvist, 1999) considered that early *Homo* with Oldowan tools had secondary access to ungulate meat and marrow through passive

scavenging of partially defleshed carcasses abandoned by their primary predators. In contrast, other researchers (e.g., Bunn and Ezzo, 1993; Domínguez-Rodrigo, 1999; Domínguez-Rodrigo and Barba, 2006; Domínguez-Rodrigo et al., 2007; Bunn and Pickering, 2010) have suggested that the hominins had primary access to fully fleshed carcasses. The reasoning of the latter is that although the extant large felids do not consume bone marrow contents, which are then available for scavengers like the hyenas, they exploit intensively the body of their prey and consequently, the flesh in the abandoned carcasses uses to be very scarce. In their view, this contradicts the interpretation of the finding of abundant cut marks related to defleshing activities in the Early Pleistocene sites as an evidence of secondary access by the hominins to these carcasses. Instead, the cut marks would reflect a primary access to the ungulate carcasses by the hominins, with carcass acquisition resulting from hunting more likely than from power, confrontational scavenging or active searching (Domínguez-Rodrigo et al., 2007, 2014). However, it is worth noting that this interpretive context is based on experimental studies performed on modern leopards and lions, which do not apply to the Early Pleistocene hominin populations with Oldowan tools that lived in Europe or in eastern and southern Africa. In the Early Pleistocene, the predator guild was dominated in the Old World by two species of sabre-tooth cats, *Megantereon whitei* and *Homotherium latidens* (Felidae, Machairodontinae), as documented in the Orce sites (Martínez-Navarro and Palmqvist, 1995, 1996; Arribas and Palmqvist, 1999; Palmqvist et al., 2007, 2011). The craniodental and postcranial anatomy of these felids, which lack of modern analogues, suggests that they were able to hunt larger ungulate prey relative to their body size compared with the living felines. For this reason, the sabre-toothed cats probably exerted a higher predation pressure on the juveniles of megafauna and exploited their prey to a lesser extent than the extant pantherine cats. This would have resulted in greater amounts of flesh abandoned in the carcasses (Martínez-Navarro and Palmqvist, 1995, 1996; Arribas and Palmqvist, 1999; Palmqvist et al., 2003, 2007, 2008a, 2008b, 2011; Ripple and Van Valkenburgh, 2010; Van Valkenburgh et al., 2016; Martín-Serra et al., 2017). Such resources would in turn be available for passive scavengers such as the giant, short-faced hyena *Pachycrocuta brevirostris,* which behaved at Orce as a strict scavenger (Palmqvist and Arribas, 2001; Palmqvist et al., 2011), and even for the hominins, as documented in FN-3 (Espigares et al., 2013).

The interpretation depicted above makes sense if we consider the limited technological skills of the Orce hominins, which make it difficult to conceive that they had a direct impact on medium-to-large and very large ungulate prey using their small Oldowan flakes (Blumenshine and Pobiner 2007). In fact, there is the proposal that the ultimate factor that allowed the first hominin dispersal in Eurasia was the arrival from Africa of *M. whitei,* a species that replaced *M. cultridens,* the sabre-tooth cat present in Eurasia before 1.8 Ma (Martínez-Navarro and Palmqvist, 1995, 1996; Arribas and Palmqvist, 1999; Palmqvist et al., 2007). This African predator had relatively enlarged sabres and showed an extreme reduction of the premolar teeth, as well as some anatomical differences in the skull related to the relative efficiency of the biting muscles at the level of the lower carnassial. Such craniodental features suggest that *M. whitei* was able to kill efficiently large ungulate prey relative to its own size. Given its dentition, extremely specialized for meat slicing, this predator only consumed soft tissues. As a result, it left variable amounts of flesh on the carcasses of the ungulates hunted and all within bone nutrients intact, which could be subsequently scavenged by the short-faced hyenas and the hominins. Moreover, the persistence of sabre-tooths in Europe helps to explain the success of the Oldowan tools in this continent, where the Oldowan/Acheulean transition took place one million years later than in Africa (Palmqvist et al., 2005). The reason is that the sharp flakes characteristic of Oldowan assemblages were fully appropriate for scavenging the carcasses partially defleshed by the sabre-tooths and the cores would have been useful for breaking the bones for accessing their marrow content (Plummer, 2004). In fact, the marked seasonality that characterized temperate Europe for most of the Pleistocene, with cooler and drier conditions than those of subtropical Africa, made the availability of scavengeable ungulate carcasses a key resource for the hominins and may have assisted the initial dispersal of *Homo* from Africa (Turner and Antón, 1998; Arribas and Palmqvist, 1999;). In contrast, the new pantherine cats that arrived in Europe during the mid-Pleistocene revolution exploited their kills more fully than the sabre-tooths, which resulted in the loss of a regular source of scavengeable carcasses for the bone-cracking hyenas and the hominins. Under these new ecological circumstances, the trophic niche that they exploited vanished. This forced the hominins toward behavioural and technological improvement, which resulted in the development of the Acheulean tools of *Homo heidelbergensis* (Arribas and Palmqvist, 1999). The destiny of the giant hyena *P.*

brevirostris, constrained by its enormous size and highly specialized anatomy, was extinction (Palmqvist et al., 2007, 2011).

In the case of the hominins that inhabited the depression of Baza and Guadix, although the scavenging of ungulate carcasses partially defleshed and abandoned by sabre-tooth cats would have enhanced their survival during Early Pleistocene times (Martínez-Navarro and Palmqvist, 1995, 1996; Arribas and Palmqvist, 1999; Martínez-Navarro, 2004, 2010; Palmqvist et al., 2005, 2007, 2011; Espigares et al., 2013; Rodríguez-Gómez et al., 2016, 2017), this does not preclude other procurement strategies (e.g., opportunistic hunting of small-sized mammals, kleptoparasitism, or even scavenging of carcasses of very large animals that died from other causes). The latter interpretation is supported by evidence of competition between *Homo* sp. and *P. brevirostris* for the exploitation of the carcass of an old individual of *Mammuthus meridionalis* in the Upper Level of FN-3. The skeleton of this elephant is dismembered and surrounded by flint flakes and coprolites, which suggests a sequential pattern of carcass consumption by hominins and hyenas (Espigares et al., 2013).

In addition, the use of a mathematical model that allows evaluating the sustainability of the community of secondary consumers, based on the biomass of primary consumers potentially available, provides relevant data on this hominin population (Rodríguez-Gómez et al., 2016). Three scenarios were modelled for estimating the population density of *Homo* sp. in the depression of Baza and Guadix: “hunter”, “scavenger” and “mixed behaviour”. The results obtained suggested that a scavenging behaviour would have been the optimal scenario for this population, as it would allow to hold 0.10-0.14 individuals per km^2 during a year, values that are close to the densities of modern hunter-gatherers (range: 0.03-0.24 individuals per km^2). In contrast, the hominin population estimated for a strict hunting behaviour, 0.09-0.10 individuals per km^2, is lower, which would compromise its viability in a basin that was relatively isolated from the nearby environments (Rodríguez-Gómez et al., 2016).

Anthropic damage of bones is documented in BL and FN-3 by the presence of cut marks, of which incisions are particularly abundant. In contrast, scrapes, sawing marks and chop marks are less recorded (see Supplementary Tables S11-S12). As explained in greater depth in the main text, the position and morphology of the cut marks relate with the activities performed during carcass processing: (i) oblique and longitudinal incisions in the limb bone midshafts relate with defleshing, while in the proximal and distal epiphyses result from disarticulation; (ii) scrapes evidence

periosteum removal; (iii) sawing marks relate to disarticulation in the metapodials and calcaneum, chop marks evidence tendon removal and incisions result from skinning and disarticulation; (iv) incisions in the jaw relate to skinning and disarticulation, while in pelvis and scapula result from defleshing; and (v) incisions on the external face of ribs and vertebral apophyses evidence defleshing, while those on the internal face of ribs and vertebral bodies point to evisceration. The predominance of cut marks resulting from defleshing and evisceration suggests that the hominins had early access to nearly intact ungulate carcasses, as would be expected in the prey of *M. whitei.* In addition, two plastron fragments of a tortoise unearthed from level BL-D show cut marks probably related with the extraction of viscera. The bones that show evidence of anthropic breakage are more abundantly represented in both assemblages than those showing cut marks. Fracture patterns evidence the systematic breakage of bones for marrow extraction.

As noted in the main text, the faunal assemblages of BL and FN-3 record no evidence of consumption of other animal resources (e.g., rodents, rabbits, birds or herpetofauna) except the two cut-marked plastron fragments from BL-D. In spite of this, the practice of a strategy based on small game is documented in many Middle and Late Pleistocene sites, although it is less frequent during the Early Pleistocene (Speth and Tchernov, 2002; Steadman et al., 2002; Blasco, 2008; Braun et al., 2010; Huguet et al., 2013; Rufá et al., 2014). If we focus on the turtles, evidence of consumption of these animals is also frequently preserved in a number of Middle and Late Pleistocene sites from Israel, Italy, Spain, or South Africa (Blasco, 2008; Blasco et al., 2013), and has been described in modern hunter-gatherer populations (Klemens and Thorbjarnason, 1995). However, apart from the two plastron fragments from Orce, there is only evidence of the exploitation of these resources during Early Pleistocene times in East Turkana (Braun et al., 2010; Archer et al., 2014) and Sima del Elefante (Blasco et al., 2011). Similarly, consumption of fish and crabs has been documented in the early Middle Pleistocene site of Gesher Benot Ya´aqov, dated at ~750 ka (Alperson-Afil et al., 2009).

Additional information on the paleodiet of ancient human populations can be retrieved from microwear analysis. In this sense, the microwear texture complexity of the hominin teeth recovered in Olduvai suggests a broader range of foods available for *Homo ergaster* than for *Australophitecus afarensis, A. africanus, H. habilis* or *Paranthropus boisei* (Ungar et al., 2012). The teeth of the Dmanisi hominins, which are

associated with stone artefacts of Oldowan tradition that are similar to those of early *Homo* in Africa, preserve a microwear pattern that falls within the range of variation for African *H. ergaster.* This suggests a similarity in their foraging behaviour (Pontzer et al., 2011). Given that the record of *Homo* sp. at Orce is limited to the deciduous molar tooth from level BL-D, no microwear analysis has been performed in the sites studied here. Although the taxonomic determination of this tooth at the species level is not possible (Toro-Moyano et al., 2013), an early *Homo* seems an appropriate candidate to inhabit the depression of Baza and Guadix in the Early Pleistocene, given the chronological proximity with the Dmanisi hominins and the presence of Oldowan lithic tools. In this sense, and carefully, the inferences derived from microwear studies performed on the Dmanisi teeth could be also extrapolated to the Orce hominins.

References cited in Supplementary Discussion S1

Aiello, L.C. & Wheeler, P. The expensive-tissue hypothesis: the brain and the digestive system in human and primate evolution. *Curr. Anthropol*. **36,** 199-221 (1995).

Alperson-Afil, N. *et al.* Spatial Organization of hominin activities at Gesher Benot Ya'aqov, Israel. *Science* **326,** 1677-1680 (2009).

Archer, W., Braun, D.R., Harris, J.W.K., McCoy, J.T. & Richmond, B.G. Early Pleistocene aquatic resource use in the Turkana Basin. *J. Hum. Evol*. **77,** 74-87 (2014).

Arribas, A. & Palmqvist, P. On the ecological connection between sabre-tooths and hominids: faunal dispersal events in the lower Pleistocene and a review of the evidence for the first human arrival in Europe. *J. Archaeol. Sci*. **26,** 571-585 (1999).

Barsky, D. *et al*. Raw material discernment and technological aspects of the Barranco León and Fuente Nueva 3 stone assemblages (Orce southern Spain). *Quat. Int.* **223-224,** 201-219 (2010).

Binford, L.R. *Bones: Ancient Men and Modern Myths* (Academic Press, 1981).

Binford, L.R. Human ancestors; changing view of their behavior. *J. Anthropol.* **4,** 292-327 (1985).

Blasco, R. Human consumption of tortoises at Level IV of Bolomor Cave (Valencia, Spain). *J. Archaeol. Sci.* **35,** 2839-2848 (2008).

Blasco, R. *et al.* Earliest evidence for human consumption of tortoises in the European Early Pleistocene from Sima del Elefante, Sierra de Atapuerca, Spain. *J. Hum. Evol.* **61,** 503-509 (2011).

Blasco, R. *et al.* Environmental availability, behavioural diversity and diet: a zooarchaeological approach from the TD10-1 sublevel of Gran Dolina (Sierra de Atapuerca, Burgos, Spain) and Bolomor Cave (Valencia, Spain). *Quat. Sci. Rev*. **70,** 124-144 (2013).

Blumenschine, R.J. *Early hominid scavenging opportunities: Implications of Carcass Availability in the Serengeti and Ngorongoro Ecosystems* (B.A.R. Int. Ser. 283, Oxford, 1986).

Blumenschine, R.J. Characteristics of an early hominid scavenging niche. *Curr. Anthropol.* **28,** 383-407 (1987).

Blumenschine, R.J. Hominid carnivory and foraging strategies, and the socioeconomic function of early archaeological sites. *Phil. Trans. R. Soc.* **334,** 211-221 (1991).

Blumenschine, R.J. Percussion marks, tooth marks, and experimental determinations of the timing of hominid and carnivore access to long bones at FLK Zinjanthropus, Olduvai Gorge, Tanzania. *J. Hum. Evol*. **29,** 21-5 (1995).

Blumenschine, R.J. & Pobiner, B.L. Zooarchaeology and the ecology of Oldowan hominin carnivory in Evolution of the human diet (ed. Ungar, P.) 167-190 (Oxford University Press, 2007).

Blumenschine, R.J., Cavallo, J.A. & Capaldo, S.D. Competition for carcasses and early hominid behavioral ecology: a case study and a conceptual framework. *J. Hum. Evol.* **27,** 197-213 (1994).

Blumenschine, R.J., Prassack, K.A., Kreger, C.D. & Pante, M.C. Carnivore tooth marks, microbial bioerosion, and the invalidation of Domínguez-Rodrigo and Barba's (2006) test of Oldowan hominin scavenging behaviour. *J. Hum. Evol*. **53,** 420-426 (2007).

Braun, D.R. *et al.* Early hominin diet included diverse terrestrial and aquatic animals, 1,95 Ma in East Turkana, Kenya. *Proc. Nat. Acad. Sci. USA* **107,** 10002-10007 (2010).

Bunn, H.T. Hunting, power scavenging, and butchering by Hadza foragers and by Plio-Pleistocene *Homo* in *Meat-Eating and Human Evolution* (eds. Stanford, C.B., Bunn, H.T.) 199-218 (Oxford University Press, 2001).

Bunn, H.T. Meat made us human in *Evolution of the Human Diet* (ed. Ungar, P.) 191-211 (Oxford University Press, 2007).

Bunn, H.T. & Ezzo, J.A. Hunting and scavenging by Plio-Pleistocene hominids: nutritional constraints, archaeological patterns, and behavioural implications. *J. Archaeol. Sci.* **20,** 365-398 (1993).

Bunn, H.T. & Pickering, T.R. Bovid mortality profiles in paleoecological context falsify hypotheses of endurance running-hunting and passive scavenging by early Pleistocene hominins. *Quat. Res*. *74,* 395-404 (2010).

Bunn, H., Pickering, T. & Domínguez-Rodrigo, M. How meat made us human: archaeological evidence of the diet and foraging capabilities of Early Pleistocene *Homo* in East Asia in *The Oxford Handbook of the Archaeology of Diet* (eds Lee-Thorp, J. & Katzenberg, M.A.) (Oxford University Press, 2017); http://www.oxfordhandbooks.com/view/10.1093/oxfordhb/9780199694013.001.0001/oxfordhb-9780199694013-e-5.

Capaldo, S.D. Experimental determinations of carcass processing by Plio-Pleistocene hominids and carnivores at FLK 22 (Zinjanthropus), Olduvai Gorge, Tanzania. *J. Hum. Evol.* **33,** 555-597 (1997).

Chivers, D.J. & Hladik, C.M. Morphology of the gastrointestinal tract in primates: Comparisons with other mammals in relation to diet. *J. Morphol.* **166,** 337-386 (1980).

Cook, J.D. 1990. Adaptation in iron metabolism. *Am. J. Clin. Nutr*. **51,** 301-308.

Domínguez-Rodrigo, M. Flesh availability and bone modifications in carcasses consumed by lions: palaeoecological relevance in hominid foraging patterns. *Palaeogeogr. Palaeoclimatol. Palaeoecol*. **149,** 373-388 (1999).

Domínguez-Rodrigo, M. & Barba, R. New estimates of tooth mark and percussion mark frequencies at the FLK Zinj site: the carnivore-hominid-carnivore hypothesis falsified. *J. Hum. Evol.* **50,** 170-194 (2006).

Domínguez-Rodrigo, M., Egeland, C.P. & Barba, R. The hunting-versus scavenging debate in *Deconstructing Olduvai: A Taphonomic Study of the Bed I Sites* (eds. Domínguez-Rodrigo, M., Barba, R. & Egeland, C.P.) 11-22 (Springer, 2007).

Domínguez-Rodrigo, M., Bunn, H.T. & Yravedra, J. A critical re-evaluation of bone surface modification models for inferring fossil hominin and carnivore interactions through a multivariate approach: Application to the FLK Zinj archaeofaunal assemblage (Olduvai Gorge, Tanzania). *Quat. Int*. **322-323,** 32-43 (2014).

Dunsworth, H. & Walker, A.C. Early genus *Homo* in *Primate Fossil Record* (ed. Hartwing, W.C.) 419-437 (Cambridge University Press, 2002).

Espigares, M.P. *et al*. *Homo* vs. *Pachycrocuta:* Earliest evidence of competition for an elephant carcass between scavengers at Fuente Nueva-3 (Orce, Spain). *Quat. Int.* **295,** 113-125 (2013).

Hardy, B.L. Climatic variability and plant food distribution in Pleistocene Europe: Implications for Neanderthal diet and subsistence. *Quat. Sci. Rev*. **29,** 662-679 (2010).

Hardy, K. et al. Diet and environment 1.2 million year ago revealed through analysis of dental calculus from Europe's oldest hominin at Sima del Elefante, Spain. Sci. Nat. 104: 2; https://doi.org/10.1007/s00114-016-1420-x (2017).

Henneberg, M., Sarafis, V. & Mathers, K. Human adaptations to meat eating. *Hum. Evol.* **13,** 229-234 (1998).

Hoberg, E.P., Alkire, N.L., de Queiroz, A. & Jones, A. Out of Africa: origins of the *Taenia* tapeworms in humans. *Proc. R. Soc. Lond. B* **268,** 781-787 (2001).

Huguet, R. *et al.* Successful subsistence strategies of the first human in south-western Europe. *Quat. Int*. **295,** 168-182 (2013).

Jiménez-Arenas, J.M., Pérez-Claros, J.A. & Palmqvist, P. Craniometric morphological patterns in hominin evolution. *Am. J. Phys. Anthropol.* **147,** 174-175 (2012).

Jiménez-Arenas, J.M., Pérez-Claros, J.A., Aledo, J.C. & Palmqvist, P. On the relationships of postcanine tooth size with dietary quality and brain volume in Primates: Implications for hominin evolution. *Biomed Res. Int.* **2014,** Article ID 406507; http://dx.doi.org/10.1155/2014/406507 (2014).

Klemens, M.W. & Thorbjarnarson, J.B. Reptiles as food resource. *Biodiv. Conserv*. **4,** 281-298 (1995).

Leonard, W.R. & Robertson, M.L. Evolutionary perspectives on human nutrition: The influence of brain and body size on diet and metabolism. *Am. J. Hum. Biol*. **6,** 77-88 (1994).

Leonard, W.R. & Robertson, M.L. On diet, energy metabolism, and brain size in human evolution. *Curr. Anthropol*. **37,** 125-129 (1996).

Mann, N. Dietary lean red meat and human evolution. *Eur. J. Nutr*. **39,** 71-79 (2000).

Martín-Serra, A., Figueirido, B. & Palmqvist, P. Non-decoupled morphological evolution of the fore- and hindlimb of sabretooth predators. *J. Anat.* **231,** 532-542 (2017).

Martínez-Navarro, B. Hippos, pigs, bovids, sabertoothed tigers, monkeys and hominids: dispersals during late Pliocene and Early Pleistocene times through the Levantine Corridor in *Human Paleoecology in the Levantine Corridor* (eds. Goren-Inbar, N. & Speth, J.D.) 37-51 (Oxbow Books, 2004).

Martínez-Navarro, B. Early Pleistocene faunas of Eurasia and hominid dispersals in *The First Hominin Colonization of Eurasia, Contributions from the Second Stony Brook Human Evolution Symposium and Workshop* (eds. Fleagle, J.G., Shea, J.J., Grine, F.E., Baden, A.L. & Leakey, R.E.) 207-224 (Springer, 2010).

Martínez-Navarro, B. Oldowan scavengers vs. Acheulian hunters: what does the faunal record say? *Glob. J. Arch. Anthropol.* 6(1): 555679; https://doi.org/10.19080/GJAA.2018.06.555679 (2018).

Martínez-Navarro, B. & Palmqvist, P. Presence of the African machairodont *Megantereon whitei* (Broom, 1937) (Felidae, Carnivora, Mammalia) in the Lower Pleistocene site of Venta Micena (Orce, Granada, Spain), with some considerations on the origin, evolution and dispersal of the genus. *J. Archaeol. Sci*. **22,** 569-582 (1995).

Martínez-Navarro, B. & Palmqvist, P. Presence of the African saber-toothed felid *Megantereon whitei* (Broom, 1937) (Mammalia, Carnivora, Machairodontinae) in Apollonia-1 (Migdonia Basin, Macedonia, Greece). *J. Archeol. Sci*. **23,** 869-872 (1996).

Martínez-Navarro, B., Espigares, M.P., Pastó, I., Ros-Montoya, S. & Palmqvist, P. Early *Homo* fossil records of Europe in *Encyclopedia of Global Archaeology* (ed. Smith, C.) 2561-2570 (Springer, 2014).

Milton, K. The critical role played by animal source foods in human (*Homo*) evolution. *J. Nutr.* **133,** 3886S-3892S (2003).

Milton, K. & Demment, M.W. Digestion and passage kinetics of chimpanzees fed high and low fiber diets and comparison with human data. *J. Nutr*. **118,** 1082-1088 (1988).

Palmqvist, P. & Arribas, A. Taphonomic decoding of the paleobiological information locked in a lower Pleistocene assemblage of large mammals. *Paleobiology* **27,** 512-530 (2001).

Palmqvist, P., Gröcke, D.R., Arribas, A. & Fariña, R. Paleoecological reconstruction of a lower Pleistocene large mammals community using biogeochemical ($\delta^{13}C$, $\delta^{15}N$, $\delta^{18}O$, Sr: Zn) and ecomorphological approaches. *Paleobiology* **29,** 205-229 (2003).

Palmqvist, P. *et al.* Réévaluation de la présence humaine au Pléistocène inférieur dans le Sud de l'Espagne. *L'Anthropologie* **109,** 411-450 (2005).

Palmqvist, P., Torregrosa, V., Pérez-Claros, J.A., Martínez-Navarro, B. & Turner, A. A re-evaluation of the diversity of *Megantereon* (Mammalia, Carnivora, Machairodontinae) and the problem of species identification in extinct carnivores. *J. Vert. Paleontol*. **27,** 160-175 (2007).

Palmqvist, P., Pérez-Claros, J.A., Gröcke, D.R. & Janis, C.M. Tracing the ecophysiology of ungulates and predator-prey relationships in an early Pleistocene large mammal community. *Palaeogeogr. Palaeoclimatol. Palaeoecol*. **266,** 95-111 (2008a).

Palmqvist, P. *et al.* Biogeochemical and ecomorphological inferences on prey selection and resource partitioning among mammalian carnivores in an early Pleistocene community. *Palaios* **23,** 724-737 (2008b).

Palmqvist, P. *et al.* The giant hyena *Pachycrocuta brevirostris:* modelling the bone-cracking behaviour of an extinct carnivore. *Quat. Int*. **243,** 61-79 (2011).

Pereira, P.M. & Vicente, A.F. Meat nutritional composition and nutritive role in the human diet. *Meat Sci.* **93,** 586-592 (2013).

Plummer, T. Flaked stones and old bones: biological and cultural evolution at the dawn of technology. *Yearb. Phys. Anthropol*. **47,** 118-164 (2004).

Pontzer, H., Scott, J.R., Lordkipanidze, D. & Ungar, P.S. Dental microwear texture analysis and diet in the Dmanisi hominins. *J. Hum. Evol*. **61,** 683-687 (2011).

Prado-Nóvoa, O., Mateos, A., Zorrilla-Revilla, G., Vidal-Cordasco, M. & Rodríguez, J. Efficiency of gathering and its archaeological implications for a European Early Paleolithic population. *J. Anthrop. Archaeol*. **45,** 131-141 (2017).

Ripple, W.J. & Van Valkenburgh, B. Linking top-down forces to the Pleistocene megafaunal extinctions. *BioScience* **60,** 516-526 (2010).

Rodríguez-Gómez, G. *et al.* On the ecological context of the earliest human settlements in Europe: Resource availability and competition intensity in the carnivore guild of Barranco León-D and Fuente Nueva-3 (Orce, Baza Basin, SE Spain). *Quat. Sci. Rev.* **134,** 69-83 (2016).

Rodríguez-Gómez, G., Palmqvist, P., Ros-Montoya, S., Espigares, M. P. & Martínez-Navarro, B. Resource availability and competition intensity in the carnivore guild of the Early Pleistocene site of Venta Micena (Orce, Baza Basin, SE Spain). *Quat. Sci. Rev.* **164,** 154-167 (2017).

Rufá, A. Blasco, R., Rivals, F. & Rosell, J. Leporids as a potential resource for predator (hominin, mammalian, carnivores, raptors): an example of mixed contribution from Level III of Teixoneres Cave (MIS 3, Barcelona Spain). *C. R. Palevol* **13,** 665-680 (2014).

Selvaggio, M.M. Evidence for a three-stage sequence of hominid and carnivore involvement with long bones at FLK Zinjanthropus, Olduvai Gorge, Tanzania. *J. Archaeol. Sci.* **25,** 191-202 (1998).

Speth, J.D. & Tchernov, E. Middle Paleolithic tortoise use at Kebara Cave (Israel). *J. Archaeol. Sci.* **29,** 471-483 (2002).

Stanford, C.B. *Meat Eating and the Origins of Human Behaviour* (Princeton University Press, 1999).

Steadman, D.W., Plourde, A. & Burley, D.V. Prehistoric butchery and consumption of birds in the Kingdom of Tonga, South Pacific. *J. Archaeol. Sci*. **2,** 571-584 (2002).

Titton, S. *et al.* Active percussion tools from the Oldowan site of Barranco León (Orce, Andalusia, Spain): The fundamental role of pounding activities in hominin lifeways. *J. Archaeol. Sci*. **96,** 131-147 (2018).

Toro-Moyano, I. *et al.* The archaic stone tool industry from Barranco León and Fuente Nueva 3 (Orce, Spain): Evidence of the earliest hominin presence in southern Europe. *Quat. Int.* **243,** 80-91 (2011).

Toro-Moyano, I., *et al.* The oldest human fossil in Europe, from Orce (Spain). *J. Hum. Evol*. **65,** 1-9 (2013).

Turner, A. & Antón, M. Climate and evolution: implications of some extinction patterns in African and European machairodontine cats of the Plio-Pleistocene. *Estud. Geol.* **54,** 209-230 (1998).

Ungar, P.S., Krueger, K.L., Blumenshine, R.J., Njau, J. & Scott, R.S. Dental microwear analysis of hominins recovered by the Olduvai Ladscape Paleonthropology Project, 1995-2007. *J. Hum. Evol*. **63,** 429-437 (2012).

Van Valkenburgh, B., Hayward, M.W., Ripple, W.J., Meloro, C. & Roth, V.L. The impact of large terrestrial carnivores on Pleistocene ecosystems. *Proc. Natl. Ac. Sci. USA* **113,** 862-867 (2016).

Wheeler, P.E. The influence of bipedalism on the energy and water budgets of early hominids. *J. Hum. Evol*. **21,** 107-136 (1991).
